# CDK6 inhibits white to beige fat transition by suppressing RUNX1

**DOI:** 10.1038/s41467-018-03451-1

**Published:** 2018-03-09

**Authors:** Xiaoli Hou, Yongzhao Zhang, Wei Li, Alexander J. Hu, Chi Luo, Wenhui Zhou, Jamie K. Hu, Stefano G. Daniele, Jinfeng Wang, Jinghao Sheng, Yongsheng Fan, Andrew S. Greenberg, Stephen R. Farmer, Miaofen G. Hu

**Affiliations:** 1 Molecular Oncology Research Institute, Tufts Medical Center, Boston, MA 02111 USA; 2 Zhejiang Chinese Medical University, Center for Analysis and Testing, 548 Bin-Wen Road, Hangzhou, 310053 P. R. China; 3 Tianjin Medical University Cancer Institute and Hospital, National Clinical Research Center of Cancer, Key Laboratory of Cancer Prevention and Therapy, Tianjin, 300060 P. R. China; 4 Department of Cancer Biology, Dana-Farber Cancer Institute and Department of Cell Biology, Harvard Medical School, Boston, MA 02115 USA; 5 Yale School of Medicine, MD program for Jamie K. Hu, MD-PhD Program for Stefano G. Daniele, 333 Cedar St, New Haven, CT 06510 USA; 6 Department of Clinical Laboratory, Linyi People’s Hospital, 27 jiefang road, Linyi, Shandong Province 276003 China; 7 Institute of Environmental Health, School of Public Health, Zhejiang University, 866 Yuhangtang Road, Hangzhou, 310058 China; 8 Obesity and Metabolism Laboratory, JM-USDA Human Nutrition Research Center, 711 Washington Street, Boston, MA 02111 USA; 9 Boston University School of Medicine, Department of Biochemistry, 72E Concord St, Boston, MA 02118 USA

## Abstract

Whereas white adipose tissue depots contribute to the development of metabolic diseases, brown and beige adipose tissue has beneficial metabolic effects. Here we show that CDK6 regulates beige adipocyte formation. We demonstrate that mice lacking the CDK6 protein or its kinase domain (K43M) exhibit significant increases beige cell formation, enhanced energy expenditure, better glucose tolerance, and improved insulin sensitivity, and are more resistant to high-fat diet-induced obesity. Re-expression of CDK6 in *Cdk6*^*−/−*^ mature or precursor cells, or ablation of RUNX1 in *K43M* mature or precursor cells, reverses these phenotypes. Furthermore, RUNX1 positively regulates the expression of *Ucp-1* and *Pgc1α* by binding to proximal promoter regions. Our findings indicate that CDK6 kinase activity negatively regulates the conversion of fat-storing cells into fat-burning cells by suppressing RUNX1, and suggest that CDK6 may be a therapeutic target for the treatment of obesity and related metabolic diseases.

## Abstract (web summary)

Beige adipocytes can arise from transdifferentiation of mature white adipocytes. Here the authors identify CDK6 as a key molecule involved in the white-to-beige adipocyte transdifferentiation and, therefore, as a regulator of organismal energy homeostasis in mice.

## Introduction

Obesity has long been known to be the most important risk factor for the development of type II diabetes and other metabolic diseases. In rodents and humans, fat is deposited as energy storage in white adipose tissue (WAT), whereas fat is consumed to produce heat in the mitochondria-rich brown adipose tissues (BAT). As a thermogenic tissue, inducible-brown adipocytes (also called beige or brite cells) are found sporadically in WAT of adult animals with similar features as classical brown adipocytes but originate from a non-myf5-derived cell lineage, likely developed from the progenitor cells residing in the stromal vascular fraction (SVF) of white adipose depots. Importantly, the activation of beige cells is associated with a protection against obesity and metabolic diseases in rodent models and correlated with leanness in human^1,2^.

Cold-induced activation of sympathetic nervous system (SNS) was previously widely believed to be the primary or only physiological signal to activate BAT/beige cells development and function, which can be mimicked by treating mice with β3-adrenergic (β3-AR) activators^3^. In addition, many genes and pathways that drive brown-fat-like thermogenesis in murine white fat have now been identified. In particular, the discovery of circulating factors such as exercise-induced cytokine (FGF-21)^4^ and mytokine (Irisin)^5^ provide a variety of promising therapeutic targets for metabolic diseases. However, while at first it may seem plausible to treat humans with β3-AR agonists, differences between rodent and human receptor physiology lead to significant off target effects^6,7^. In turn, this has halted the development of β3-AR agonists as a viable treatment for obesity-related metabolic diseases. Nevertheless, the challenges facing long-term maintenance of regular exercise and cold exposure abound, due to natural human tendency for thermal comfort as well as modern-day time constraints. Thus, in face of the imminent epidemic, there is an urgent need for a new therapeutic target to attain weight control and to improve the obesity-related metabolic profile.

Cyclin-dependent Kinase 6 (CDK6) plays an important role in proliferation and differentiation^8^. Although regulation of cell cycle is deemed to be the primary function of CDK6^9^, it also acts in a cell cycle-independent manner, as evidenced by binding and promoting the degradation of RUNX1 (also known as AML1)^10^. As a member of the Runt-related transcription factors, RUNX1 recognizes a specific DNA sequence, which upon binding, activates or represses the transcription of several downstream genes^11^. However, the molecular roles that CDK6 and RUNX1 play in obesity and its associated metabolic diseases remains largely unexplored.

Employing our defined *Cdk6* mouse models and in vitro differentiation assay, we have observed that the loss of either CDK6 (*Cdk*^*−/−*^*)* or its kinase domain (*K43M*) results in white fat browning, enhanced energy expenditure, improved glucose tolerance and insulin sensitivity, and resistance to high-fat diet (HFD)-induced obesity (DIO). Importantly, recovery of CDK6 expression or ablation of *Runx1* reversed this metabolic phenotype. Therefore, targeting CDK6 may be a therapeutic strategy to treat obesity and its related metabolic diseases.

## Results

### CDK6 responds to changes in nutritional status

To explore the function of CDK6 in adiposity, we first determined the expression of CDK6 protein in adipose tissues. In male and female mice, CDK6 was expressed in both BAT and WAT, e.g. inguinal WAT (iWAT) and epididymal WAT (eWAT), respectively (Supplementary Fig. 1a). Similar to leptin, a protein produced by fatty tissue and considered to regulate fat storage, CDK6 mRNA and protein were selectively up-regulated in iWAT and eWAT but not in BAT in C57BL/6J mice under an HFD compared to the mice under a normal chow diet (NCD) (Supplementary Fig. 1b, c). Furthermore, CDK6 protein level was also higher in mice that fasted overnight compared to the mice on an NCD (Supplementary Fig. 1d). Taken together, these data indicate that CDK6 responds to changes in nutritional status.

### *K43M* mice are resistant to HFD-induced obesity

To understand the functional relevance of CDK6 in obesity and its related diabetes, we utilized CDK6 knockout and knockin mice^12,13^. We produced genetically distinct animals by introducing a LoxP-flanked transcriptional STOP cassette (LSL cassette) into intron 1 of the *Cdk6* gene adjacent to the intact or mutant exon1. In the presence of the LSL cassette, CDK6 expression is prevented, which results in a null allele named: *Cdk6*^*−/−*^ *(KO)*, or *WT-LSL*, or *K43M-LSL* (Supplementary Fig. 1e). Upon excision of the cassette by germline expression of *Cre* recombinase (Nestin-CRE), the CRE-reactivated wild-type (referred to as *WT* hereafter) or the mutant alleles express either WT or mutant protein (Supplementary Fig. 1f), respectively, from the endogenous locus with intact regulatory controls, specifically, the knockin mutant with a catalytically inactive kinase domain, CDK6^K43M^ (K43M)^13^. The significance of this particular mutant is that Lys 43 residue is highly conserved across all eukaryotic kinases and represents one of three residues forming a triad of catalytic residues involved in ATP phosphate orientation and Mg2+ coordination^14,15^. Therefore, expression of the K43M mutation is a powerful genetic model equivalent to pharmacological inhibition of CDK6 kinase activity.

**Fig. 1:**
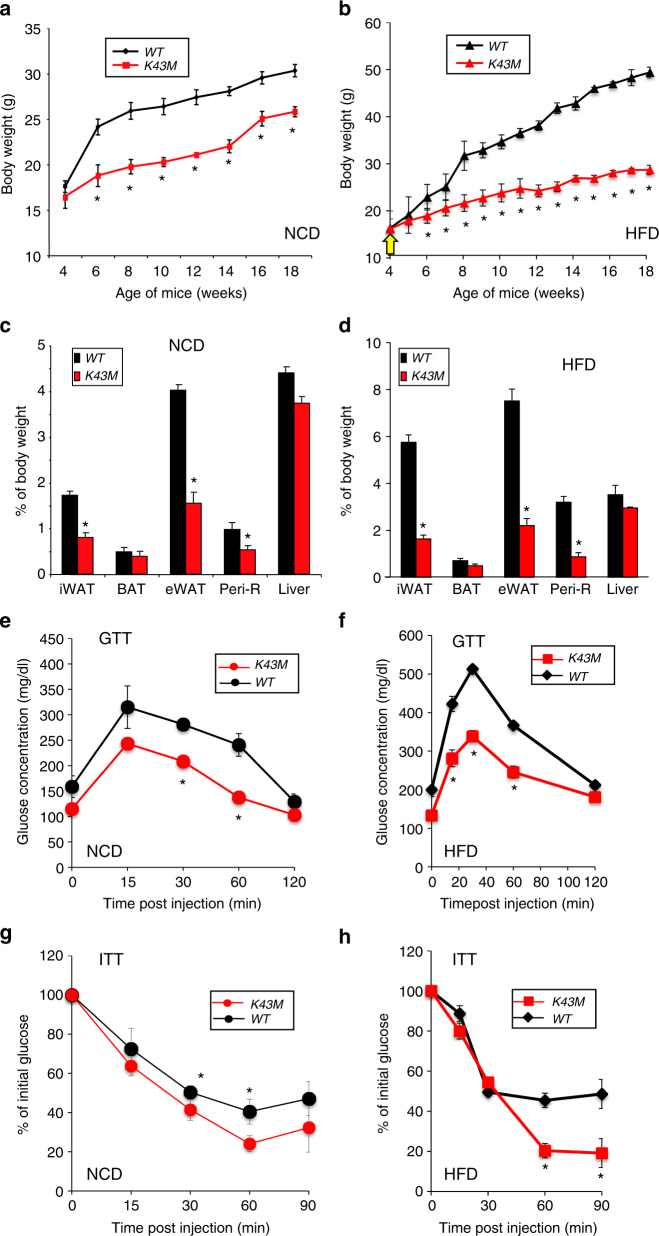
Loss of CDK6 kinase activity in mice leads to an improved metabolic profile. **a**, **b** Body weight of age-matched male mice on NCD (**a**) or HFD (**b**) for a 14-week observation time. HFD commenced at 4 weeks of age. **c**, **d** Mass of various fat pads was normalized to body weight of male mice on NCD (**c**) or HFD (**d**) at 18 weeks of age. No significant changes were observed in the masses of BAT and livers between *WT* and *K43M* mice under NCD and HFD. **e**, **f** GTT after 14 weeks on NCD (**e**) or HFD (**f**). **g**, **h** ITT after 14 weeks on NCD (**g**) or HFD (**h**). For **a**–**h**, data shown are mean ± S.E. (*n* = 10 for each group), *p < 0.05, *t*-test, vs *WT*. See also Supplementary Figs. 2 and 3

To investigate if loss of CDK6 kinase activity alters the metabolic physiology of the mice, we first subjected wild-type (*WT*) and *K43M* mice to either NCD or an HFD (starting at 4-week-old) over a 14-week period. Both male and female (data not shown) *K43M* mice displayed increased resistance to weight gain under NCD and HFD (Fig. 1a, b, Supplementary Fig. 2a). To help elucidate the underlying mechanism governing protection against DIO, we isolated different fat pads and tissues of 12-week-old (Supplementary Fig. 2c, d) and 18-week-old (Fig. 1c, d) mice, respectively, and measured their weighs relative to the whole-body weights of the mice. We observed that *K43M* mice had significantly decreased fat pad mass (~1.5- to 4-fold reduction compared with *WT*) in all WAT depots analyzed, including iWAT, eWAT, and perirenal (Peri-R) compartments, which were consistent with decreased adiposity assessed using NMR (Supplementary Fig. 2a, b). By contrast, interscapular BAT depots were not significantly different in weight between *WT* and *K43M* mice (Fig. 1c, d and Supplementary Fig. 2e). In addition, no significant mass changes were observed in the liver of *WT* and *K43M* mice under NCD and HFD (Fig. 1c, d). Collectively, these results show that loss of CDK6 kinase activity systemically in mice protects from DIO.

### *K43M* mice exhibited improved GTT and ITT

We next evaluated whether *K43M* mice exhibited an improved metabolic profile by conducting intraperitoneal glucose- and insulin-tolerance tests (IP-GTT and IP-ITT, respectively). Compared to their *WT* littermates, *K43M* mice fed on both NCD and HFD displayed rapid clearance and resulted in lower blood glucose concentrations after glucose (Fig. 1e, f) and insulin (Fig. 1g, h) injection, suggesting that genetic disruption of CDK6 improves blood glucose tolerance and insulin sensitivity.

### Loss of CDK6 kinase activity leads to white fat browning

**Fig. 2:**
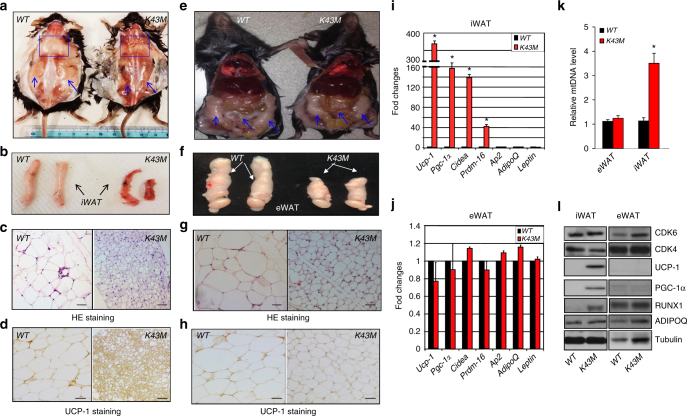
Loss of CDK6 kinase activity in mice induced white fat browning. **a**, **e** Appearance of male posterior-subcutaneous WAT (sWAT) (**a**), and dorsal view of NCD-fed *WT* and *K43M* mice (**e**), emphasized with blue squares and arrows. **b**, **f** Appearance of a close view of the iWAT (**b**) and eWAT (**f**) from the mice indicated. **c**, **g** Representative light microscopic images of H&E-stained sections of iWAT (*n* = 6) and eWAT (*n*–6) from male mice indicated (scale bars: 100 μm). **d**, **h** Representative images of UCP-1 immunohistochemical staining (*n* = 6) of iWAT and eWAT from mice indicated at 18 weeks of age (scale bars: 100 μm). **i**, **j** Relative mRNA expression levels of BAT-specific markers (*Ucp-1*, *Pgc-1α*, *Cidea*, and *Prdm16)* and WAT-specific markers (*Ap2*, *adiponectin-AdipoQ*, and *Leptin*) of iWAT (**i**) and eWAT (**j**) tissues from *WT* and *K43M* mice. Data shown are mRNA fold change normalized to the control *WT*, which is arbitrarily set to 1 unit. **k** Relative expression levels of mtDNA of eWAT and iWAT from *WT* and *K43M* mice. Data shown are fold change of mtDNA compared to control. For **i** and **j**, *p < 0.05, vs *WT* (*n* = 6), *t*-test. **i** Immunoblots of the indicated protein levels in iWAT and eWAT from 50 μg of cell lysates of *WT* and *K43M* mice at 18 weeks of age. α-Tubulin is used as an internal loading control. See also Supplementary Figs. 2 and 3

Under NCD, both male (Fig. 2) and female (data not shown) *K43M* mice were found on dissection to have browner appearance in various fat pads, such as posterior-subcutaneous (Fig. 2a) and iWAT (Fig. 2b), than their *WT* counterparts. The size of adipocytes in *K43M* mice were smaller (Fig. 2c) but the brown-type features of iWAT were evidenced by presence of much more abundant multilocular UCP-1^+^ beige adipocytes in iWAT (Fig. 2d). By contrast, despite reduced fat pad mass and smaller cell sizes, *K43M* and *WT* mice had similar appearance with comparable UCP-1^+^ staining in eWAT (Fig. 2e–h), suggesting that the underlying mechanisms governing the homeostasis of sWAT and visceral adipose tissue (VAT) of *K43M* mice were different. Consistently, BAT-specific genes *Ucp-1*, *Pgc-1α*, *Cidea*, *and Prdm16* were expressed at significantly higher levels in iWAT (Fig. 2i) but not in eWAT (Fig. 2j) of *K43M* mice compared to *WT*. In contrast, the WAT-specific genes including *Ap2*, *Adiponectin (AdipoQ)*, and *Leptin* were expressed at comparable levels in iWAT and eWAT (Fig. 2i, j) from both *K43M* and *WT* mice. Furthermore, iWAT but not eWAT of *K43M* mice had higher expression of mitochondria DNA (mtDNA) than that of *WT* mice (Fig. 2k). In parallel with mRNA levels, UCP-1 and PGC-1α, two factors contributing to leanness in various mouse models^16^, were selectively expressed at higher levels in iWAT, but not eWAT of *K43M* mice as compared to *WT* mice (Fig. 2l, Supplementary note), suggesting enhanced beige cells in iWAT depots. However, mass and cell size in BAT (Supplementary Fig. 2e–g) were comparable between *WT* and *K43M* mice. Moreover, although BAT-specific genes were also marginally, yet significantly, increased in the BAT (~1.8- to 2.3-fold) (Supplementary Fig. 2h) of *K43M* mice, no significant differences were noted in CDK6, PGC-1a, and UCP-1 protein levels between *WT*-BAT and *K43M*-BAT mice under NCD (Supplementary Fig. 2i). Taken together, these data suggest that increased white fat browning, rather than an overall reduced WAT adipogenesis, may underlie the reduced fat pad mass in *K43M* mice under NCD.

Under the same experimental condition, we also observed that the RUNX1 protein, a downstream target of CDK6^17,18^ was increased in iWAT but not in eWAT of *K43M* adipocytes in comparison to *WT* controls (Fig. 2l). However, the levels of *Runx1* mRNA were comparable (Supplementary Fig. 2j), thereby ruling out a transcriptional effect of K43M on RUNX1 levels. Together with the known essential functions of RUNX1 in many biological programs related to development^19^, we hypothesized that RUNX1 may be involved in the process of white fat browning in iWAT of *K43M* mice.

Under HFD, a ~3- to 80-fold induction of BAT-specific genes (Supplementary Fig. 3g) and a ~1.5- to 3-fold suppression of WAT-specific genes (Supplementary Fig. 3h) were observed in iWAT of *K43M* mice vs those in *WT* mice. RUNX1 protein was also increased in the iWAT of *K43M* compared to *WT* controls (Supplementary Fig. 3i). Together, these data suggest that under NCD, the gross reduction of sWAT pad mass in *K43M* mice resulted from a partial replacement of white fat with beige cells. Under HFD, however, both increased white fat browning and reduced WAT adipogenesis in *K43M* mice might account for the reduced adiposity in *K43M* mice.

### *K43M* mice have increased energy expenditure

**Fig. 3:**
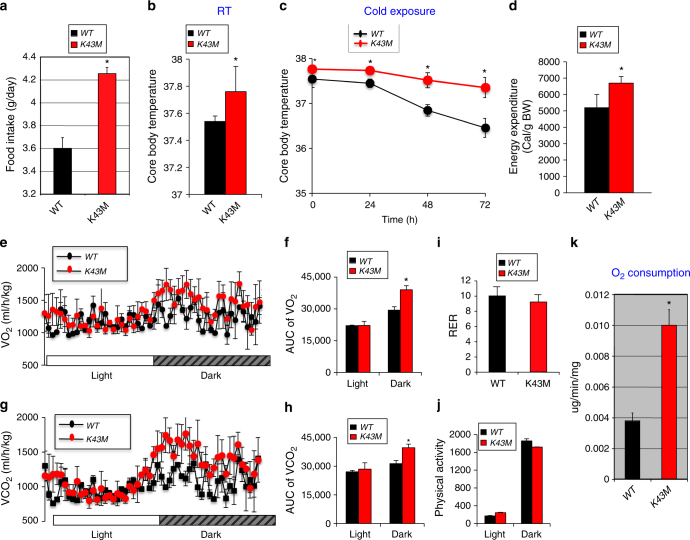
Loss of CDK6 kinase activity in mice leads to increased food intake, body temperature, adaptability to cold exposure and energy expenditure. **a** Bar graphs demonstrating food intake of age-matched male *WT* and *K43M* mice at 18 weeks of age. **b**, **c** Core body temperature of male mice at room temperature (**b**, RT) or at 4 °C (**c**) for up to 72 h (*n* = 6 per group). **d** Energy expenditure (EE) was calculated based on the formula below: EE = (3.815 + 1.232 × RER) x VO_2_/lean mass (g). For **a**–**d**, data are expressed as mean ± S.E., *p < 0.05, vs *WT*, *t*-test. **e**–**h**, Oxygen consumption (VO_2_) (e, f) and CO_2_ production (VCO_2_) (g, h) from metabolic cages of male *WT* and *K43M* mice on NCD (*n* = 6) in 12 h light and dark phases. VO_2_ and VCO_2_ were normalized by lean mass. *p < 0.05, vs *WT*, log-rank tests. **i** Bar graphs displaying mean Respiratory Exchange Ratios (RER) over 24-h period. RER was calculated as the volume of CO_2_ vs the volume of oxygen (VCO_2_/VO_2_). **j** Physical activity of male *WT* and *K43M* mice in the periods of 12 h light and 12 h dark phase. **k** Ex vivo oxygen consumption of iWAT homogenates from different *WT* and *K43M* mice. Data are expressed as mean ± S.E., *p < 0.05, vs *WT*, *t*-test

A physiological hallmark of beige cells is their highly active metabolism coupled to thermogenesis, similar to that featured in classical brown fat cells^20^. To determine if increased browning in *K43M* mice correlates with increased caloric intake as well as thermogenesis, we examined the food consumption and body temperature of mice under different conditions. As expected, *K43M* mice had significantly more food intake (4.25 ± 0.05 g/day) than *WT* mice (3.6 ± 0.09 g/day) (Fig. 3a) and elevated body temperature compared to *WT* mice at room temperature (Fig. 3B, RT). Robust differences were apparent after cold exposure: the body temperature of *WT* mice was significantly lower than their *K43M* counterparts by 0.28 °C (1-day, *p* = 0.03), 0.67 °C (2-day, *p* = 0.02), and 0.90 °C (3-day, *p* = 0.03), respectively (Fig. 3c). Upon dissection, *K43M* mice were found to have browner appearance in iWAT than their *WT* counterparts but comparable appearance on BAT (Supplementary Fig. 4a–c). Compared with *WT*-iWAT, the magnitude of the increment in *Ucp-1* gene and protein expression in *K43M*-iWAT was much smaller (Supplementary Fig. 4d, e) than those in RT (Fig. 2i, l and Supplementary Fig. 4g). It is noteworthy that RUNX1 protein but not the mRNA level in *K43M-iWAT* was also enhanced under cold exposure (Supplementary Fig. 4d, e). However, the expressions of *Cdk6* gene and protein were comparable between *WT* and *K43M* mice. These results indicate that increased white fat browning in *K43M* mice is reflected by a significantly higher body temperature and thus more adaptive than *WT* mice to cold-induced thermogenesis.

To further investigate the mechanism underlying the difference in reduced adiposity in 18-week-old *K43M* mice, we monitored the metabolic activities of these mice by using metabolic cages. NCD-fed *K43M* mice had increased energy expenditure during the three-day observation period (Fig. 3d), as indicated by significantly greater O_2_ consumption (Fig. 3e, f, dark phase) and greater CO_2_ production (Fig. 3g, h, dark phase) than *WT* controls during the nocturnal phase. Importantly, there is no measurable difference in the respiration exchange ratio (RER) and physical activity between *WT* and *K43M* mice (Fig. 3i, j). These data indicate that the reduced adiposity in *K43M* mice might be caused by an increase in energy expenditure rather than in physical activity.

Lean mass is mainly composed of skeletal muscle, a major contributor to resting and exercise-induced energy expenditure^21^. Undetectable CDK6 protein in skeletal muscle (Supplementary Fig. 1a) prompted us to focus on the contribution of fat tissues to energy expenditure. For this purpose, we have measured energy expenditure directly on iWAT but not the skeletal muscle of different mutant mice by using the Clark electrode to further confirm that the adipose tissues indeed contribute to the increased energy expenditure in *K43M* mice. When measuring the respiration rate of freshly minced fat tissue, the O_2_ consumption of iWAT was increased significantly in *K43M* mice compared to that in *WT* mice (Fig. 3k), indicating that the adipose tissues indeed contribute to the increased energy expenditure in *K43M* mice. However, it is unknown at present whether CDK6 contributes to the energy expenditure in skeletal muscle.

Collectively, these results demonstrate that loss of CDK6 kinase activity in mice results in increased thermogenic function through elevation of energy expenditure, which may account for more adaptability in response to a cold exposure and for protection against HFD-induced obesity.

### SNS activation is not the major cause of white fat browning

The brown appearance of WAT in *K43M* mice (germline) could, in principle, be mediated by activation of β–adrenergic receptors^22^, targets of the catecholamines. The loss of CDK6 function in adipocytes could result in a generalized stress response (increased adrenergic stimulation) that then indirectly results in browning of iWAT, along with increased energy expenditure and all of the other phenotypes observed in *Cdk6* mutant mice.

In rodents, exposure to cold stimulates the sympathetic nerve system (SNS) to biosynthesize the tyrosine hydroxylase (TH)^23^, the rate-limiting enzyme in the synthesis of noradrenaline^24^, and then release more adrenaline^25,26^, which increases *Ucp-1* expression in WAT^27^. To examine directly if white fat browning is due to activation of SNS, firstly, we exposed mice to RT or cold environments (4 °C) with or without inhibition of noradrenaline synthesis. To achieve this, mice were treated with α-methyl-*p*-tyrosine (α-MPT), a competitive specific inhibitor of TH^23,24^, for 24 h^25^. We then examined the effects of the treatments on *Ucp-1* expression in iWAT. Consistent with previous studies^23,25,27^, cold stimulation markedly enhanced TH and *Ucp-1* in *WT-*iWAT (Supplementary Fig. 4f, g), but this enhancement was clearly reversed with α-MPT administration in *WT* mice. In contrast, the magnitudes of increment in TH and *Ucp-1* gene expression in *K43M*-iWAT after cold stimulation were much smaller (Supplementary Fig. 4f, g) than those in *WT-*iWAT, and the expression of UCP-1 protein was comparable between *K43M* mice with or without α-MPT administration (Supplementary Fig. 4h) at RT. These finding indicate that SNS involvement in the regulation of UCP-1 expression in *WT* mice, but not the major determinant in *K43M* mice. Therefore, the WAT browning observed in *K43M* mice is not chiefly mediated by activation of β-adrenergic system.

### Cell-autonomous effect of CDK6 on white fat browning

The brown appearance of WAT in *K43M* mice could be a direct conversion from mature white adipocytes. To address this question, we crossed *Cdk6*^*−/−*^ *or K43M-LSL* mice with Adiponectin-Cre (Adipoq-Cre) mice (Supplementary Fig. 5a), which express CRE in mature adipocyte^28^ within WAT and BAT. The resultant mice are named *WT-Aand K43M-A* for re-expression of CDK6 or expression of K43M proteins in mature adipocytes. DNA recombination and *Cre* expression in adipocytes of the resultant mice were confirmed by PCR (Supplementary Fig. 5b)^13^. Immunoblot analysis demonstrated that the levels of CDK6/K43M expression in *WT-A and K43M-A* mice were about 50–60% of WT (Supplementary Fig. 5c), which suggests that Adipoq-Cre elicited partially penetrant recombination in mature adipocytes and/or no observable recombination in the SVF^29^ of white adipose depots where the beige cell progenitors reside. Consistently, no CDK6 protein in other tissues such as thymocytes (Supplementary Fig. 5d) was detected in *WT-A* and *K43M-A* mice. In line with previous studies^29,30^, *WT-A and K43M-A* mice were born at the expected Mendelian frequency, and display fertility with normal development. They have comparable body lengths as *WT* and *KO* mice (Supplementary Fig. 5e). Similar to *K43M* mice, *KO* and *K43M-A* mice ate more than *WT* mice, whereas *WT* and *WT-A* mice had similar food intake each day (Supplementary Fig. 5f).

**Fig. 4:**
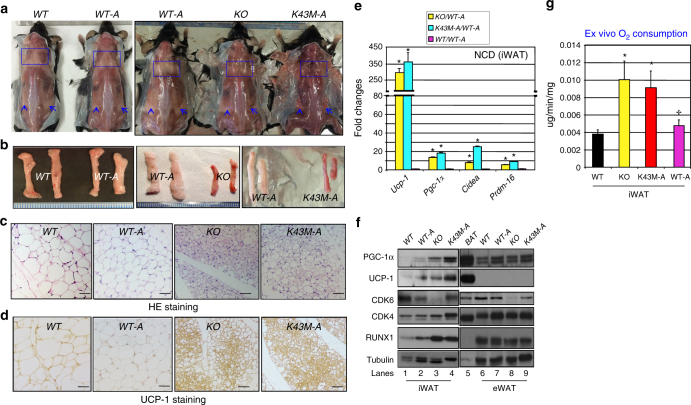
Re-expression of CDK6 in mature adipocytes of *KO* mice reverses white fat browning. Loss of kinase activity in mature adipocytes preserves the effect of loss of kinase activity in germline on white fat browning. **a** Appearance of male (18 weeks of age) posterior-sWAT of NCD-fed *WT*, *WT-A*, *KO*, and *K43M-A*, emphasized with blue squares and arrows. **b** Appearance of isolated iWAT from the mice indicated in **a**. **c** Representative light microscopic images of H&E-stained sections of iWAT (*n* = 6) from male (18 weeks of age) mice indicated in **a** (scale bars: 100 μm). **d** Representative images of UCP-1 staining (*n* = 6) of iWAT from mice indicated at 18 weeks of age (scale bars: 100 μm). **e** Relative mRNA expression levels of BAT-specific markers (*Ucp-1*, *Pgc-1α*, *Cidea*, and *Prdm16)* of iWAT under NCD. Data shown are mRNA fold change normalized to their respective controls, *WT* or *WT-A*. *p < 0.05, *n* = 6, *t*-test, comparing experimental group vs its control. **f** Immunoblots of the indicated protein levels in iWAT and eWAT from 50 μg of cell lysates. Twenty micrograms of cell lysates of BAT was used as a positive control for UCP-1 and PGC-1α, and α-tubulin was utilized as an internal loading control. **g** Ex vivo oxygen consumption of iWAT homogenates from mice. Data are expressed as mean ± S.E., *p < 0.05, *n* = 6, vs *WT*, *t*-test. ^✢^*p* < 0.05, *n* = 6 vs *KO*, *t*-test. See also Supplementary Figs. 3 and 5

We found that re-expression of CDK6 in mature adipose cells of *WT-A* mice reversed the browning of WAT, indicating that CDK6 negatively regulates white fat browning in a cell-autonomous manner, whereas expression of the inactive kinase (*K43M-A*) in mature adipocytes on a null background preserves the *K43M* or null phenotype (Fig. 4a–d). Consistently, the BAT-specific genes were expressed at higher levels in iWAT of *KO *or *K43M-A* mice compared to controls under both NCD and HFD, which is reflected, to a similar degree, as *K43M* vs *WT* (Fig. 4e and Supplementary Fig. 3g). A number of these genes are also modestly but significantly increased in eWAT (Supplementary Fig. 3e) of *KO* and *K43M-A* mice. However, immunoblot analysis confirmed the higher expression of UCP-1, PGC-1α, and RUNX1 (Fig. 4f, lanes 1–4, Supplementary note) in iWAT but not in eWAT of *KO* and *K43M-A* mice (Fig. 4f, lanes 6–9, Supplementary note). By contrast, *Ap2* and *AdipoQ* were comparable between *KO/K43M-A* and *WT-A* mice (Supplementary Fig. 3f), as in *K43M*-iWAT (Fig. 2i) under NCD, but reduced significantly under HFD (Supplementary Fig. 3h). *Leptin*, however, was expressed at significantly lower levels in iWAT of *KO/K43M-A* under both NCD and HFD (Supplementary Fig. 3f, h). All gene expression levels were comparable between *WT-A* and *WT* cells (Fig. 4e and Supplementary Fig. 3e–h). Importantly, adipose-specific re-expression of CDK6 significantly reversed the increased oxygen consumption of iWAT observed in *KO* mice, whereas *K43M-A* mice recapitulated *KO* mice in terms of oxygen consumption (Fig. 4g). Therefore, re-expression of CDK6 in mature adipocytes can reverse the phenotypes observed in *KO* mice, and adipose-specific expression of K43M can recapitulate the phenotypes observed in *KO/K43M* mice.

### Cell-autonomous effect of CDK6 on GTT and ITT

**Fig. 5:**
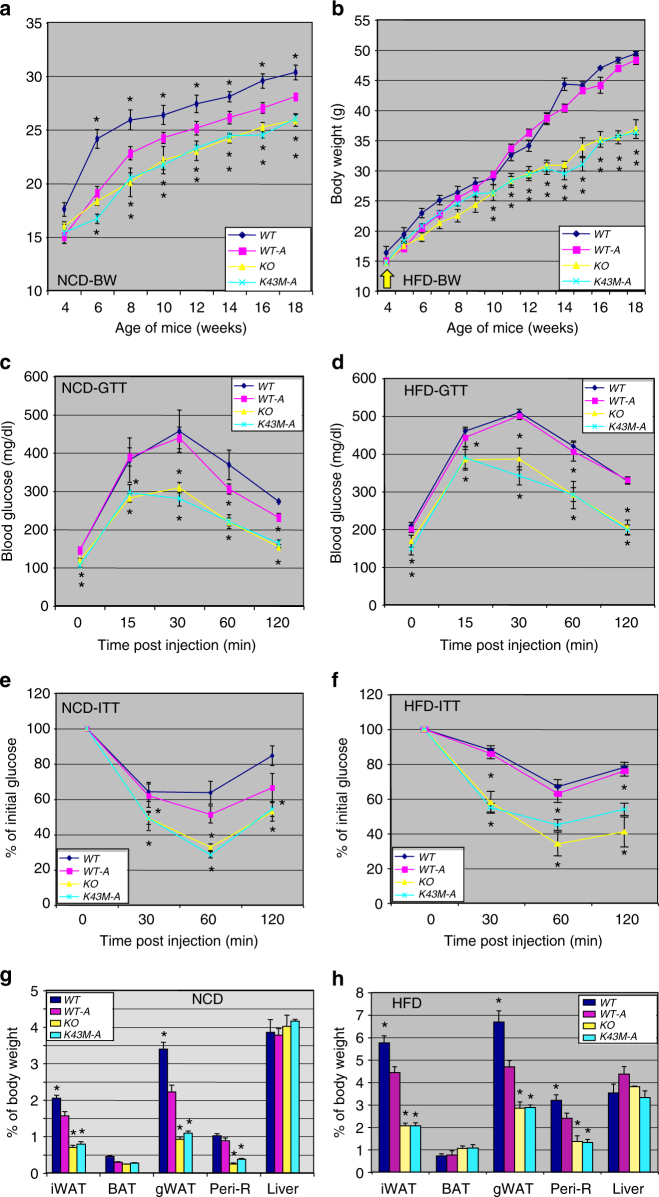
Re-expression of CDK6 in mature adipocytes reversed the beneficial metabolic effects observed in *KO* mice, whereas re-expression of kinase dead CKD6 recapitulates this metabolic profile. **a**, **b** Body weight of age-matched male mice on NCD (**a**) or HFD (**b**) for 14-week observation time. HFD started at age of 4 weeks. **c**, **d** GTT after 18 weeks on NCD (**c**) or HFD (**d**). **e**, **f** ITT after 18 weeks on NCD (**e**) or HFD (**f**). **g**, **h** Mass of various fat pads was normalized to body weight of male mice on NCD (**g**) or HFD (**h**) at age of 18 weeks. No significant changes were observed in the masses of BAT and livers in those mice under NCD and HFD. For **a**–**h**, data are expressed as mean ± S.E. (*n* = 10 for each group), *p < 0.05, *t*-test, vs *WT-A*. See also Supplementary Fig. 3

To check whether browning of WAT in *K43M-A* mice elicits the same advantageous metabolic effects as *K43M* mice, we fed age-matched male *K43M-A* together with *WT*, *KO*, and *WT-A* mice a NCD or a HFD for 14 weeks starting at 4 weeks of age. *KO and K43M-A* mice had similar body weight under both diets, exhibiting significantly reduced body weight on both NCD (Fig. 5a) and HFD (Fig. 5b and Supplementary Fig. 3b), compared to *WT-A* mice. Re-expression of CDK6 in mature adipocytes in *WT-A* mice restored the body weight completely under HFD but only partially under NCD compared to *WT* mice during the observation period (Fig. 5a, b), suggesting that the effects of CDK6 on the development of other tissues such as thymocytes, hematopoietic stem progenitors^12,13^ and progenitors of adipocytes might account for the baseline body weight difference between *WT* and WT-A mice fed with NCD.

Similar to *K43M* mice with a germline mutation, *K43M-A and KO* mice displayed better glucose tolerance (Fig. 5c, d), more sensitivity to insulin (Fig. 5e, f), and drastically reduced fat pad masses (Fig. 5g, h and Supplementary Fig. 3d), ranging from ~2- to 3.6-fold reduction in different fat pads, compared to those of *WT-A* mice. Consistent with reduced weight gain observed under NCD, *WT-A* had slightly but significantly reduced fat pad masses in various depots compared to those of *WT* mice, ranging from ~1.3- to 1.6-fold reduction, suggesting re-expression of CDK6 in mature adipocytes only partially rescued the defect of *KO* mice in WAT development.

Together, these data suggest that adipose-specific re-expression of CDK6, but not K43M, reverses the beneficial metabolic effects observed in *KO* mice, indicating that the CDK6 kinase activity regulates metabolic homeostasis in a cell-autonomous manner. But it remains to be seen if only adipocyte-specific loss of CDK6 or kinase activity imparts the observed effects on the metabolic profile.

### Loss of CDK6/kinase activity increases beige cells in vitro

**Fig. 6:**
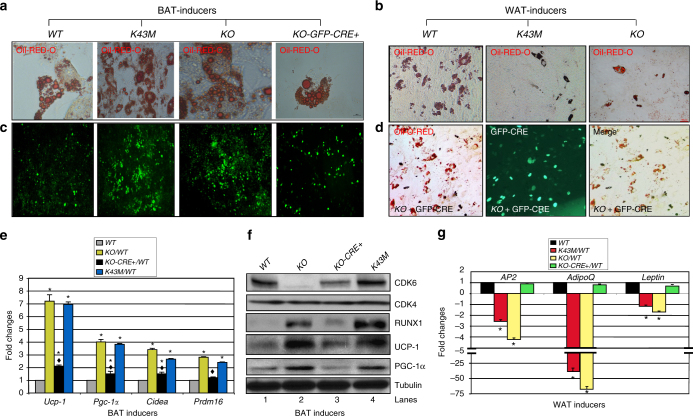
Re-expression of CDK6 in precursors of *KO* cells inhibits differentiation towards brown-like adipocytes. **a**, **b**, **d** Representative images of Oil Red O staining of differentiated SVF cells in the presence of BAT inducers (**a**) or WAT inducers (**b**, **d**). **c** Representative images of MitoTracker green staining of differentiated SVF cells from iWAT with BAT inducers. MitoTracker green is a cell-permeable probe to label mitochondria. e, **g** Relative mRNA levels of BAT-specific markers (**e**) and WAT markers (**g**) in the differentiated SVF cells in the presence of BAT or WAT inducers, respectively. Data shown are mRNA fold change normalized to the relative *WT* controls, which was arbitrarily defined as 1 unit, *p < 0.05, vs *WT*, *t*-test (*n* = 6). **f** Immunoblots of the indicated protein levels in differentiated cells from 50 μg of cell lysates. We used α-tubulin as an internal loading control

The brown appearance of WAT in *K43M* mice (germline) could be a direct (cell-autonomous) consequence of loss of CDK6 kinase activity on specification and/or differentiation from beige precursors. In order to confirm that the observed phenotypes are indeed due to cell-autonomous adipocyte browning, we sought to determine if ablation of CDK6 kinase activity in vitro promotes differentiation towards brown-like adipocytes using mouse primary adipose derived stem cells (ADSCs) from the SVF of adipose tissue. The confluent primary ADSCs isolated from iWAT of *WT*, *KO*, or *K43M* mice were stimulated with brown- or white fat inducers for 7 days. With BAT inducers, ADSCs derived from *K43M* or *KO* mice turned into brown-like adipocytes (Fig. 6a, e, f), but had defects to differentiate into WAT in the presence of WAT inducers (Fig. 6b, g), as demonstrated by Oil Red O (ORO) staining (Fig. 6a, b, d) as well as the increased BAT markers and reduced expression of WAT markers (Fig. 6e–g). Consistently, *KO* and *K43M* cells contain a greater number of mitochondria than *WT* cells, as determined by MitoTracker staining, a cell-permeable probe used to label mitochondria (Fig. 6c). Re-expression of CDK6 in *KO* cells (Fig. 6f, lane 3) after retroviral infection with MigR1-GFP-CRE^17^ reversed advantageous in BAT-like differentiation (Fig. 6a, c, e, f) and the defect in WAT differentiation (Fig. 6b, d, g). Together, these data suggested that the absence of CDK6 kinase activity restricts precursors from executing WAT adipogenic programs in a cell-autonomous manner, and CDK6 may be essential for WAT vs BAT lineage commitment.

### CDK6 inhibits white fat browning by suppressing RUNX1 in vivo

To understand the cellular and molecular mechanisms through which CDK6 negatively regulate white fat browning, we first employed loss of function studies in vivo because of the inverse relationship between CDK6 kinase activity and RUNX1 protein abundance (Figs. 2l, 4f, 6f, and Supplementary Fig. 3i). Consistent with previous observation^10^, CDK6 does interact with RUNX1 (Supplementary Fig. 6a, b), and phosphorylation of RUNX1 on serine 303 was reduced in K43M cells (Supplementary Fig. 6a, b). Thus, the overabundance of RUNX1 in the absence of CDK6 protein or kinase activity may be due to its reduced phosphorylation status by CDK6 and subsequent reduced degradation.

**Fig. 7:**
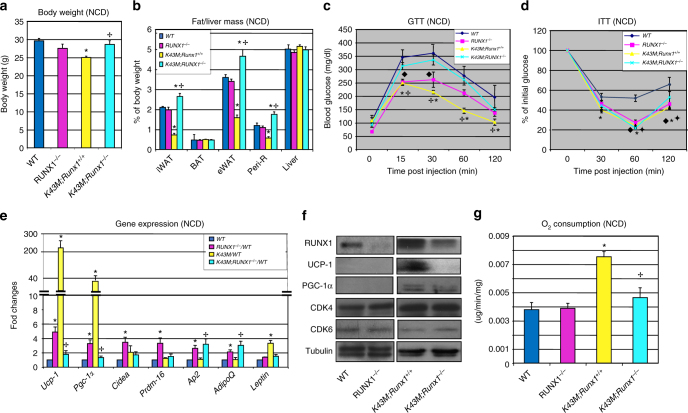
CDK6 inhibits white fat browning by suppressing RUNX1. **a** Body weight of age-matched male mice (18-week-old) on NCD. *p < 0.05, (*n* = 6), vs *WT*, *t*-test. ^✢^*p* < 0.05, *t*-test, *K43M;Runx1*^*−/−*^ vs *K43M*. **b** Mass of various fat pads was normalized to body weight of male mice on NCD at 18 weeks of age. No significant changes were observed in the masses of BAT and livers among mice indicated under NCD. Data shown are mean ± S.E. (*n* = 10 for each group), *p < 0.05, *t*-test, vs *WT*. ^✢^*p* < 0.05, *t*-test, *K43M;Runx1*^*−/−*^ vs *K43M*. **c** GTT after 18 weeks on NCD. **d** ITT after 18 weeks on NCD. For **c** and **d**, *n* = 10 for each group, *p < 0.05, *t*-test, *K43M *vs *WT*, ^♦^*p* < 0.05, *t*-test, *Runx1*vs *WT*, ^✢^*p* < 0.05, *t*-test, *K43M;Runx1*^*−/−*^ vs *K43M*, ^◆^*p* < 0.05, *t*-test, *K43M;Runx1*^*−/−*^ vs *WT*. **e** Relative mRNA expression levels of BAT-specific markers and WAT-specific markers of iWAT tissues. Data shown are fold changes of mRNA normalized to the control *WT*, which is arbitrarily set to 1 unit. *p < 0.05 (*n* = 6), vs *WT* control, *t*-test. ^✢^*p* < 0.05, *t*-test, *K43M;Runx1*^*−/−*^ vs *K43M*. **f** Immunoblots of the indicated protein levels in iWAT from 50 μg of cell lysates of the mice indicated at 18 weeks of age. α-Tubulin is used as an internal loading control. **g** Ex vivo oxygen consumption of iWAT homogenates from mice indicated. Data are expressed as mean ± S.E., *p < 0.05, vs *K43M*, *t*-test. ^✢^*p* < 0.05, *t*-test, *K43M;Runx1*^*−/−*^ vs *K43M*. See also Supplementary Figs. 6 and 7

To genetically ablate RUNX1 in *WT* and *K43M* mice, we crossed *Runx1*^fl/fl^ and *K43M;Runx1*^fl/fl^ with Adipoq-Cre mice to remove RUNX1 in mature adipocytes. The presence of DNA recombination of *Cdk6* alleles, *Cre* expression, *Runx1* alleles and Floxed-*Runx1* alleles, and deleted Floxed-*Runx1* alleles were confirmed by PCR^12,13,31^ in adipocytes of the resultant mice (Supplementary Fig. 6c). Immunoblot analysis demonstrated that the *Runx1*^*−/−*^ and *K43M;Runx1*^*−/−*^ mice still expressed low levels of RUNX1 (Fig. 7f), which may result from partially penetrant recombination and/or residual RUNX1 protein from progenitors in the adipocytes.

Compared to *K43M* mice under both NCD (Fig. 7) and HFD *(*Supplementary Fig. 7), loss of *Runx1* in the *K43M *mice resulted in significantly more body weight and fat masses (Fig. 7a, b and Supplementary Fig. 7a, b), reversed white fat browning (Supplementary Fig. 6d–g), and reduced glucose tolerance (Fig. 7c and Supplementary Fig. 7c). Interestingly, insulin sensitivity was reduced in *K43M;Runx1*^*−/−*^ *mice* under HFD (Supplementary Fig. 7d) but not under NCD (Fig. 7d). Consistent with observed phenotypes, *K43M;Runx1*^*−/−*^ mice had reduced expression of BAT-specific genes and proteins (Fig. 7e, f, Supplementary note) increased WAT-specific genes (Fig. 7e and Supplementary Fig. 7e), and reduced O_2_ consumption (Fig. 7g). In comparing *WT* and *K43M;Runx1*^*−/−*^ mice, the similar expression levels of BAT-specific genes and parallel increase in expression of *Ap2* and *AdipoQ* (Fig. 7e) may in part explain why *K43M;Runx1*^*−/−*^ mice have greater fat pad masses than *WT* mice.

In contrast, loss of *Runx1* in *WT* mice did not affect the gross and histological appearance of subcutaneous fat (Supplementary Fig. 6d–g), fat pad masses (Fig. 7b and Supplementary Fig. 7b), cell sizes (Supplementary Fig. 6f), and UCP-1 staining (Supplementary Fig. 6g). Similarly, ablation of *Runx1* in *WT* mice also led to increased BAT-specific genes in iWAT (Fig. 7e). However, these increases are counteracted at least in part by increased WAT-specific genes in iWAT (Fig. 7e), thus maintaining the homeostatic WAT development. Together, these data demonstrate that RUNX1 mediated the effects of K43M on the regulation of white fat browning.

### Loss of RUNX1 in *K43M* precursors reverse white fat browning

**Fig. 8:**
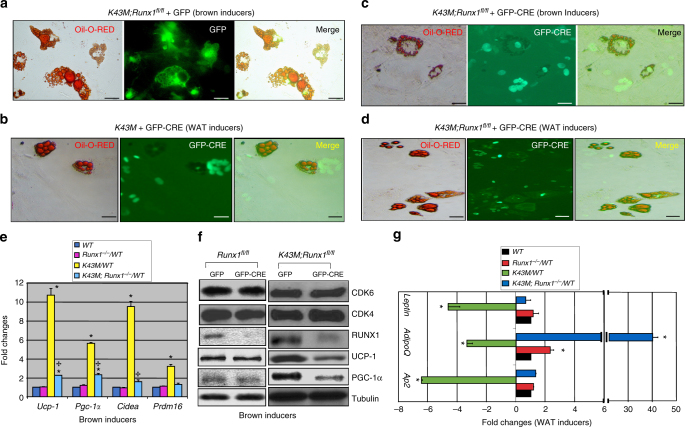
Ablation of RUNX1 in *K43M* precursors inhibits differentiation towards brown-like adipocytes but promotes differentiation towards white adipocytes. **a–d** Fluorescent photomicrographs of differentiated cells from *K43M* cells (*K43M;Runx1*^*fl/fl*^ + GFP or *K43M* + GFP-CRE) (**a**, **b**) or *K43M;Runx1*^*−/−*^ (*K43M;Runx1*^*fl/fl*^ + GFP-CRE)(cd) in the presence of BAT (**a**, **c**) or WAT inducers (**b**, **d**). Red fluorescence indicates the Oil Red O staining. Green fluorescence indicates the expression of GFP/GFP-Cre. The yellow fluorescence indicates merged images from red and green fluorescence. Scale bar: 50 μm. **e**, **g** Relative mRNA levels of BAT or WAT markers in differentiated cells in the presence of BAT (**e**) or WAT inducers (**g**). Data shown are the mRNA fold change of different mutants normalized to their relative *WT* controls, which was arbitrarily defined as 1 unit, *p < 0.05, vs WT, *t*-test (*n* = 6). ^✢^*p* < 0.05, *t*-test, *K43M;Runx1*^*−/−*^ vs *K43M*. **f** Immunoblots of the indicated protein levels in the differentiated cells from 50 μg of cell lysates after 7 days in the presence of BAT inducers. α-Tubulin is used as an internal loading control. See also Supplementary Figs. 8 and 9

In vitro, after retroviral infection with MigR1-GFP (GFP) or MigR1-GFP-CRE^17^ (GFP-CRE), reduction of RUNX1 in the cells was confirmed by using western blotting (Supplementary Fig. 8a, b, and Fig. 8f). Compared to those *K43M* cells (*K43M;Runx1*^*fl/fl*^ + GFP or *K43M* + GFP-CRE) (Fig. 8a, b, e, f), *K43M;Runx1*^*−/−*^ (*K43M;Runx1*^*fl/fl*^ + GFP-CRE) exhibited reduced BAT differentiation with BAT inducers (Fig. 8c, e, f), but increased WAT differentiation with WAT inducers (Fig. 8d, g). By contrast, *Runx1*^*−/−*^(*Runx1*^*fl/fl*^ + GFP-CRE) cells and *WT* cells had comparable capacity of differentiation (Supplementary Fig. 8c, d). Together, these data suggest that loss of RUNX1 in *K43M* precursors reversed the phenotypes observed in *K43M* mutant cells such that the cells displayed increased WAT formation while simultaneously decreasing BAT formation.

### RUNX1 binds to mouse *Ucp-1* and *Pgc-1α* promoters

To understand the mechanism underline RUNX1-mediated browning, we employed a chromatin immunoprecipitation (CHIP) assay to determine if RUNX1 binds to RUNX1-consensus-binding sites in the mouse *Ucp-1* and *Pgc-1α* genes. Bioinformatic (Matinspector) analysis (Supplementary Fig. 9a, b) identified three core consensus sequences (TGTGNNN, whereby NNN can stand for TTT or TCA) on the proximal promoter regions of *Pgc-1α* and *Ucp-1* genes, respectively. We used both RUNX1- and CDK6-specific antibodies followed by quantitative PCR (qPCR) assay utilizing primers flanking the core consensus sequence (Supplementary Table 1 and Supplementary Fig. 9a, b). ChIP analysis demonstrated that RUNX1 bound specifically to the sites of *Pgc-1α* and *Ucp-1 genes* in *WT* pre-adipocytes and differentiated adipocytes, and this binding was significantly increased in *K43M* cells (Supplementary Fig. 9c–e). These results indicate that increased RUNX1 protein and DNA-binding activity are integral for upregulation of BAT markers in *K43M* mice.

## Discussion

The main finding of this study is that CDK6 is a crucial negative regulator of white to beige fat transition. We provided biochemical and physiological data demonstrating that CDK6, and more specifically its kinase domain, is critical for adipocyte biology and metabolism. We report that genetic inhibition of CDK6 signaling in mice promotes the expression of genes critical for beige cell biogenesis and is associated with browning of WAT, which together impart an advantageous metabolic profile. Our data revealed a previously unknown function of CDK6 in fat metabolism, and suggest that inhibitors of the kinase activity of CDK6 would be beneficial in reversing deleterious effects of metabolic syndromes, and therefore serve as a novel therapeutic target in the treatment of obesity and its related metabolic diseases.

We found that CDK6 deficient-mediated WAT browning is due to cell-autonomous effect of CDK6 loss on fat precursor differentiation and conversion of mature white adipocytes, but not due to SNS activation, supporting previous hypothesis that beige cell development is caused by both de-novo differentiation of precursor cells^32^ and by transdifferentiation of existing white adipocytes^33^. The browning-mediated by loss of CDK6 kinase activity is physiological relevance since loss of CDK6/kinase activity in mice resulted in weight loss, enhanced energy expenditure, improved glucose tolerance, and insulin sensitization. Conversely, re-expression of CDK6 in mature adipocytes (*WT-A)* reverses the beneficial metabolic effects observed in *KO* mice, indicating that the CDK6 kinase activity regulates metabolic homeostasis in a cell-autonomous manner, although whether this is due to the appearance of brite fat or whether other mechanisms play a role is difficult to discern at the moment.

We also found that the effects of CDK6 in conversion of fat-storing cells into fat-burning cells is independent of pRB activity and, therefore, most likely independent of cell cycle control. pRB has been show to play multiple roles in metabolic functions including repressing *PGC-1*α expression^34^ and suppressing peroxisome proliferator-activated receptor gamma subunit^35^. Mouse embryo fibroblasts deficient in pRB did not undergo adipose conversion in response to standard adipogenic inducers, but rather differentiated into brown adipose^36^. We demonstrate that CDK6 has pRB-independent role in adipogenesis by regulating RUNX1, one of the downstream effectors of CDK6^10^ known to be frequently mutated in human leukemia and play a role in hematopoiesis^37^. To the best of our knowledge, our findings demonstrate, for the first time, a molecular interplay between CDK6 and RUNX1 and in the regulation of white fat browning. This mechanism appears, at least in part, to be due to a CDK6 kinase-mediated suppression of RUNX1 that normally promotes *Ucp-1* and *Pgc-1α* expression. Genetic deletion of *Runx1* in *K43M* mice rescues most of the phenotype observed in mutant mice in vivo and restores WAT differentiation capacity of *K43M* precursors in vitro, demonstrating a downstream role for RUNX1 in CDK6-mediated white fat browning.

**Fig. 9:**
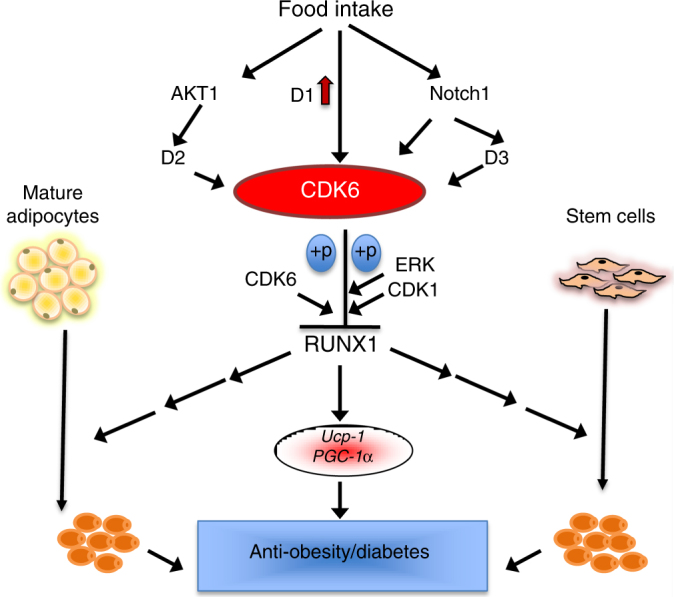
A working model of the role of CDK6 in negative regulation of white fat browning by suppressing RUNX1. In response to food intake, increased expression of cyclin D1^38^ and CDK6 leads to activation of CDK6 (Supplementary Fig. 1). In addition, the canonical cascade of events is initiated, including the activation of Notch1^39^ and AKT1^40^. Notch1 activates CDK6 via upregulation of CDK6 and/or by increased cyclin D3 protein^41^. AKT1 activates CDK6 by stabilizing cyclin D2^42^. Phosphorylation of RUNX1 by CDK6 and other kinases such as ERK and CDK1^10^ promotes RUNX1 proteolytic degradation^10^, resulting in a reduction of RUNX1 recruitment to the proximal promoter regions of *Ucp-1* and *Pgc-1α* (Supplementary Fig. 9), subsequently leading to reduced level of BAT-specific protein expression. In the absence of CDK6 protein/kinase activity, RUNX1 is stabilized, BAT-specific protein expression is increased, and obesity and its related metabolic diseases are suppressed

Mechanistically, our results suggest that CDK6 is integrated into the white fat browning pathways and therefore serve as a sensor and an effector of cellular energy status as shown in Fig. 9. In response to food intake, increased expression of cyclin D1^38^ and CDK6 (Supplementary Fig. 1) leads to activation of CDK6. In addition, the canonical cascade of events is initiated, including the activation of Notch1^39^ and AKT1^40^. Notch1 activates CDK6 via upregulation of CDK6 and/or by increased cyclin D3 protein^41^. AKT1 activates CDK6 by stabilizing cyclin D2^42^. Phosphorylation of RUNX1 by CDK6 and other kinases such as ERK and CDK1^10^ promotes RUNX1 proteolytic degradation^10^, resulting in a reduction of RUNX1 recruitment to the proximal promoter regions of *Ucp-1* and *Pgc-1α* (Supplementary Fig. 9), subsequently leading to reduced level of BAT-specific protein expression. In the absence of CDK6 protein/kinase activity, RUNX1 is stabilized, BAT-specific protein expression is increased, and obesity and its related metabolic diseases are suppressed.

Therapeutically, our data also show that CDK6 could be better therapeutic target in metabolic diseases than CDK4, a cyclin-dependent kinase that has been implicated in metabolism by phosphorylating PGC-1α^43^ and insulin receptor substrate 2^44^. However, loss of CDK4 has been shown to result in insulin-deficient diabetes due to a severe decrease in β-cell growth^45^. In contrast, loss of function of CDK6 enhances both energy expenditure and improves glucose tolerance and insulin sensitivity, but has no obvious effect on pancreas^46^. Thus, inhibition of CDK6 kinase activity might open up new perspectives in the control of metabolic diseases and could be a better therapeutic target in obesity-related metabolic diseases such as type II diabetes.

Overall, our findings reveal that CDK6 kinase activity functions as a potent regulator of white to beige cell transition, providing a new therapeutic target for pharmacological intervention aimed at combatting the imminent obesity epidemic and its related metabolic diseases.

## Methods

### Mice

#### Generation of different *mature adipocytes-specific* mutant mice and *K43M;Runx1*^*−/−*^ mice

We backcrossed mice bearing the *WT-LSL and K43M-LSL* (we made) alleles eight times to C57BL/6. Mature adipocyte-specific *WT-A/K43M-A* mutant mice, *Runx1*^*−/−*^ mice, and *K43M;Runx1*^*−/−*^ mice were produced by crossing *WT-LSL*, *K43M-LSL*, *Runx1*^*fl/fl*^ (Jackson lab, stock number: 010673) and *K43M;Runx1*^*fl/fl*^ alleles with Adipoq-Cre mice (Jackson lab, stock number: 010803), respectively. All experiments were performed according to the guidelines of the Institutional Animal Care and Use Committee of Tufts University.

### GTT and ITT tests

The experimental procedures described previously^47^. We used mice at age of 12–18 weeks.

### Body composition

Body composition was analyzed by NMR.

### Energy expenditure, locomotor activity, and food intake

Energy expenditure, locomotor activity, and food intake were determined by metabolic cages using a TSE system. Both NMR and TSE system are in the Adipocyte Biology and Nutrient Metabolism Core of BNORC. Oxygen consumption (VO_2_), carbon dioxide production (VCO_2_), and spontaneous motor activity during 3 consecutive days were measured and normalized to lean mass.

### Ex vivo oxygen consumption

Direct ex vivo tissue oxygen consumption was measured by a Clark electrode (Strathkelvin Instruments in Dana-Farber). Freshly isolated fat tissue was minced in respiration buffer (1.5 mM pyruvate, 25 mM glucose, 2% bovine serum albumin) and placed in electrode chambers. The O_2_ consumption rate was normalized to tissue weight.

### In vitro differentiation assay

The primary ADSCs from the SVF of iWAT of *WT*, *KO*/*K43M*, *Runx1*^*fl/fl*^, and *K43M;Runx1*^*fl/fl*^ mice were isolated as previously described^48^. To re-express CDK6 or delete RUNX1, *KO/Runx1*^*fl/fl*^/*K43M;Runx1*^*fl/fl*^ were infected with MigR1-GFP-CRE^17^. In order to induce differentiation, adipocyte progenitor cells were left for a further 48 h after reaching confluence, then induced with white^49^ or brown/beige adipocytes inducers^50^ for 7 days. On day 8, accumulation of lipid-containing cells was detected by Oil red O staining as described^51^. Fluorescent photomicrographs of differentiated cells were presented. Red fluorescence indicates the Oil-Red-O staining. Green fluorescence indicates the expression of GFP/GFP-Cre. Yellow fluorescence indicates the merged Red and Green fluorescence. In a subset of experiments, the differentiated cells were also stained with MitoTracker, a cell-permeable probe used to label mitochondria. Adipogenic gene expression of WAT and BAT markers was analyzed by reverse transcription PCR (RT-PCR) and immunoblotting.

### Chromatin immunoprecipitation

We performed ChIP according to the protocol described by Upstate Biotechnology. Pre-adipocytes and differentiated cells were treated with formaldehyde and immunoprecipitated with antibody against RUNX1 (*rabbit anti-RUNX1 antibody*, Cat. ab23980*)*, CDK6 (C-21, Santa Cruz), and normal rabbit IgG, respectively. The immunoprecipitates were eluted and reverse crosslinked for 5 h at 65 °C. qPCR was performed with the primers listed in Supplemental Table 1 (site 1-site 3 of *Ucp-1* and *Pgc-1α*). For the internal control, genomic DNA was extracted from pre-adipocytes or differentiated cells and 1% (input) of the extracted DNA was amplified with the same primers listed in Supplemental Table 1.

#### Immunoblotting, IP-Western, and RNA purification

Mice were sacrificed by CO_2_ and spinal dislocation, and tissue samples were removed, clamp-frozen in liquid nitrogen, and stored at −80 °C until being processed as described previously^12,13^. Antibodies used in this study included CDK6 (ab3126, mouse monoclonal antibody, Abcam), CDK6 (C-21, Santa Cruz, 1:1000 dilution), RUNX1 (Ab23980, Rabbit polyclonal antibody, 1:1000 dilution, Abcam), CDK4 (C-22, Santa Cruz, 1:1000 dilution), RUNX1-phospho-S303 (Ab55308, Abcam, 1:1000 dilution), α-Tubulin (Sigma, mouse monoclonal, 1:2000 dilution), UCP-1 (AB23841, Abcam, 1:1000 dilution), PGC-1α (SC13067, H-300, Santa Cruz, 1:1000 dilution), Anti-Tyrosine Hydroxylase antibody (ab75875, 1:1000 dilution).

For Supplementary Fig. 6a, we immunoprecipitated RUNX1 with antibody (AB54869-100, mouse monoclonal antibody, Abcam) from extracts of adipose tissues of iWAT and probed the blots with the indicated antibodies RUNX1-phospho-S303 (Ab55308, Abcam), CDK6 (C-21, Santa Cruz), and total RUNX1 (Ab23980, Rabbit polyclonal antibody, Abcam). For Supplementary Fig. 6b, we immunoprecipitated CDK6 with antibody (ab3126, mouse monoclonal antibody, Abcam) from extracts of adipose tissues of iWAT and probed the blots with the same antibodies described above as for Supplementary Fig. 6a. For detecting CDK6, we used CDK6 (C-21, Santa Cruz). The antibody used for CHIP assay was obtained from Abcam (rabbit anti-RUNX1 antibody, Cat. ab23980).

#### Quantitative real-time PCR (RT-PCR)

The experimental procedures are same as those described previously^47^. The 36B4 gene, encoding an acidic ribosomal phosphoprotein P0 (RPLP0), was used as an invariant internal control. The Primer sequences for genotyping of *Cdk6* mutant^12,13^ and *Runx1* mutant mice^31^, for RT-PCR of brown fat genes including *Ucp-1*, *Pgc-1α*, *Cidea*, and *Prdm16*(ref. ^47^) are same as those described previously. Other primer sequences for RT-PCR or for qPCR are listed in Supplemental Table 1.

### Statistical methods and statistical analysis

For most experiments, the sample size was chosen based on expected differences between experimental and control groups in order to provide adequate power to detect a significant difference specifying *α* = 0.05, two-tailed testing, and power (=1−*β*) of 80%, using commercially available software packages (Statistical Solutions nQuery Advisor; http://www.statsol.ie/nquery/nquery.htm). All data are expressed as means ± S.E. We calculated statistical significance using Student’s *t*-test, or log-rank tests, with *P* < 0.05 considered significant.

### Data availability

All relevant data are available from the authors.

## Electronic supplementary material

Supplementary Information
