# Supplementary material for: CDK6 inhibits white to beige fat transition by suppressing RUNX1
Source: Nat Commun. 2018 Mar 9;9:1023. doi: 10.1038/s41467-018-03451-1 (PMC5845007; doi:10.1038/s41467-018-03451-1)
Supplement: Supplementary file 1 — Supplementary Information [file 41467_2018_3451_MOESM1_ESM.pdf]

(A) SUPPLEMENTARY FIGURES

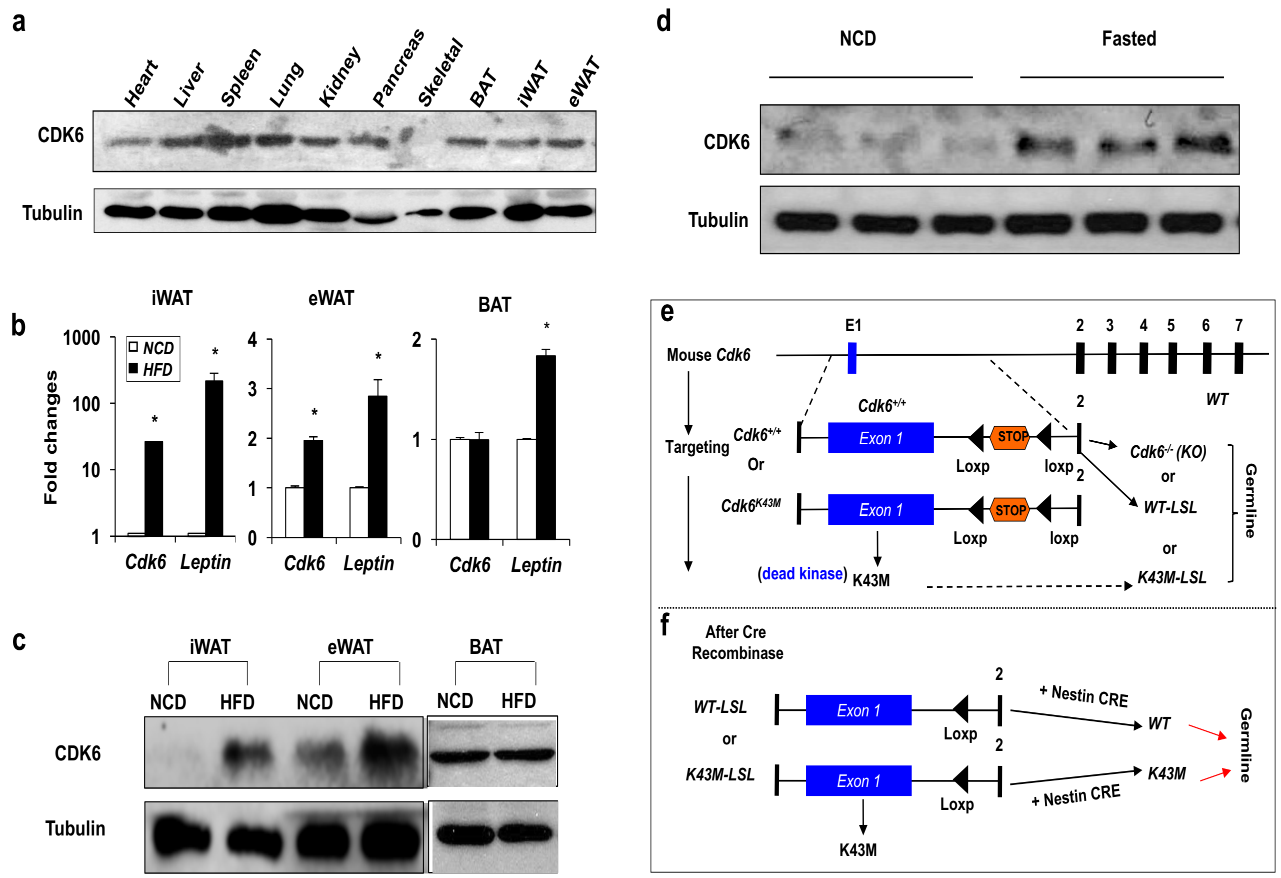

Supplementary Figure 1. CDK6 responses to changes in nutritional status. **(a)** Tissue distribution of CDK6 was determined by immunoblotting analysis of equal amount (50μG) of cell lysates from different tissues. BAT: brown adipose tissue; iWAT: inguinal white fat; eWAT: epididymal white fat. **(b)** Expression level of *Cdk6* and *Leptin* mRNA in iWAT, eWAT, and BAT from *WT* male mice following 14-weeks of NCD or HFD starting at 4 weeks of age. Data shown are mRNA fold change in mice under HFD normalized to the control mice under NCD*,* which is arbitrarily set to 1 unit. **p* < 0.05, (n=6), *vs* NCD, t-test. **(c)** CDK6 protein levels of iWAT, eWAT, and BAT under either NCD or HFD. **(d)** CDK6 protein levels of iWAT under either NCD or Fasted. For (**a-d)**, α-Tubulin was used as an internal control. (**e**) *Cdk6*$^{-/-}$ mice were made by introducing a LoxP-flanked transcriptional STOP cassette (LSL) into intron 1 of the *Cdk6* gene adjacent to the intact (*Cdk6*$^{+/+}$) / mutant exon 1 (for instance, dead kinase mutation: *K43M*). In the presence of the LSL cassette, CDK6 expression is prevented, resulting in a null allele

(named *Cdk6*$^{-/-}$, or *KO*, *or WT-LSL,* or *K43M-LSL*). (**f**) Upon excision of the cassette by Nestin-CRE, which is expressed in germline, the CRE-reactivated *WT*/mutant alleles express CDK6/mutant protein from the endogenous locus with intact regulatory control. The knock-in mutants include a catalytically inactive kinase (K43M: dead kinase), mimicking the outcome of pharmacological inhibition of CDK6.

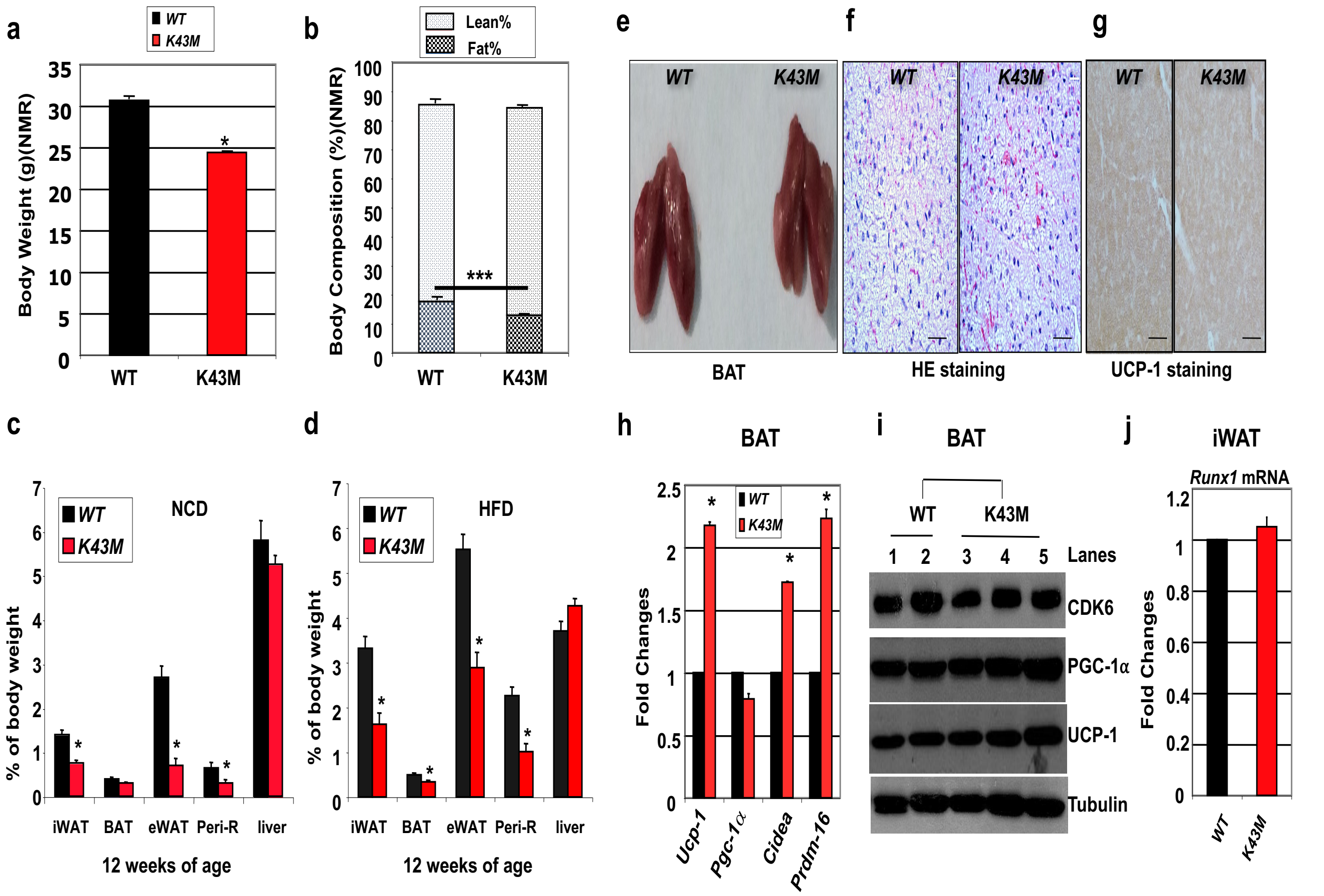

Supplementary Figure 2. Effects of loss of CDK6 kinase activity on body composition, fat mass (12 weeks of age) BAT, and *Runx1* mRNA. (**a**) Body weight of 18 weeks old male mice, *p < 0.05, vs *WT* mice (n=6), t-test. (**b**) Body compositions of 18 week-old male mice. Body composition was determined by NMR analysis. *WT* mice had more contribution from fat (17.67 ± 1.21% BW for *WT vs* 12.89 ± 0.56% of BW for *K43M*), but less contribution from lean mass (67.71 ± 1.48% of BW for WT mice vs 71.37 ± 1.01% BW for K43M). ***p < 0.05 for fat mass, vs *WT* mice (n=6), t-test. (**c,d**) Mass of various fat pads was normalized to body weight of male mice on NCD (**c**) or HFD (**d**) at 12 weeks of age. No significant changes were observed in the masses of BAT and livers between *WT* and *K43M* mice under NCD and

HFD. For c and d, data shown are mean ± S.E. (n=10 for each group), *p < 0.05, t-test, vs *WT*. (**e**) Appearance of male BAT from the mice indicated. (**f**) Representative light microscopic images of H&E-stained sections of BAT (n=6) from male *WT* and *K43M* mice at 18 weeks of age (scale bars: 100 μm). (**g**) Representative images of UCP-1 staining (n=6) of BAT from male *WT* and *K43M* mice at 18 weeks of age (scale bars: 100 μm). **(h, j)** Relative mRNA expression levels of BAT-specific markers (*Ucp-1*, *Pgc-1α, Cidea, and Prdm16 in BAT* (**h**) and *Runx1* (**j**) of iWAT from *WT* and *K43M* mice. Data shown are fold changes of mRNA normalized to the control *WT,* which is arbitrarily set to 1 unit. *p < 0.05, (n=6), t-test. (i) CDK6, PGC-1 α, and UCP-1 protein levels of BAT under NCD. α-Tubulin was used as an internal control.

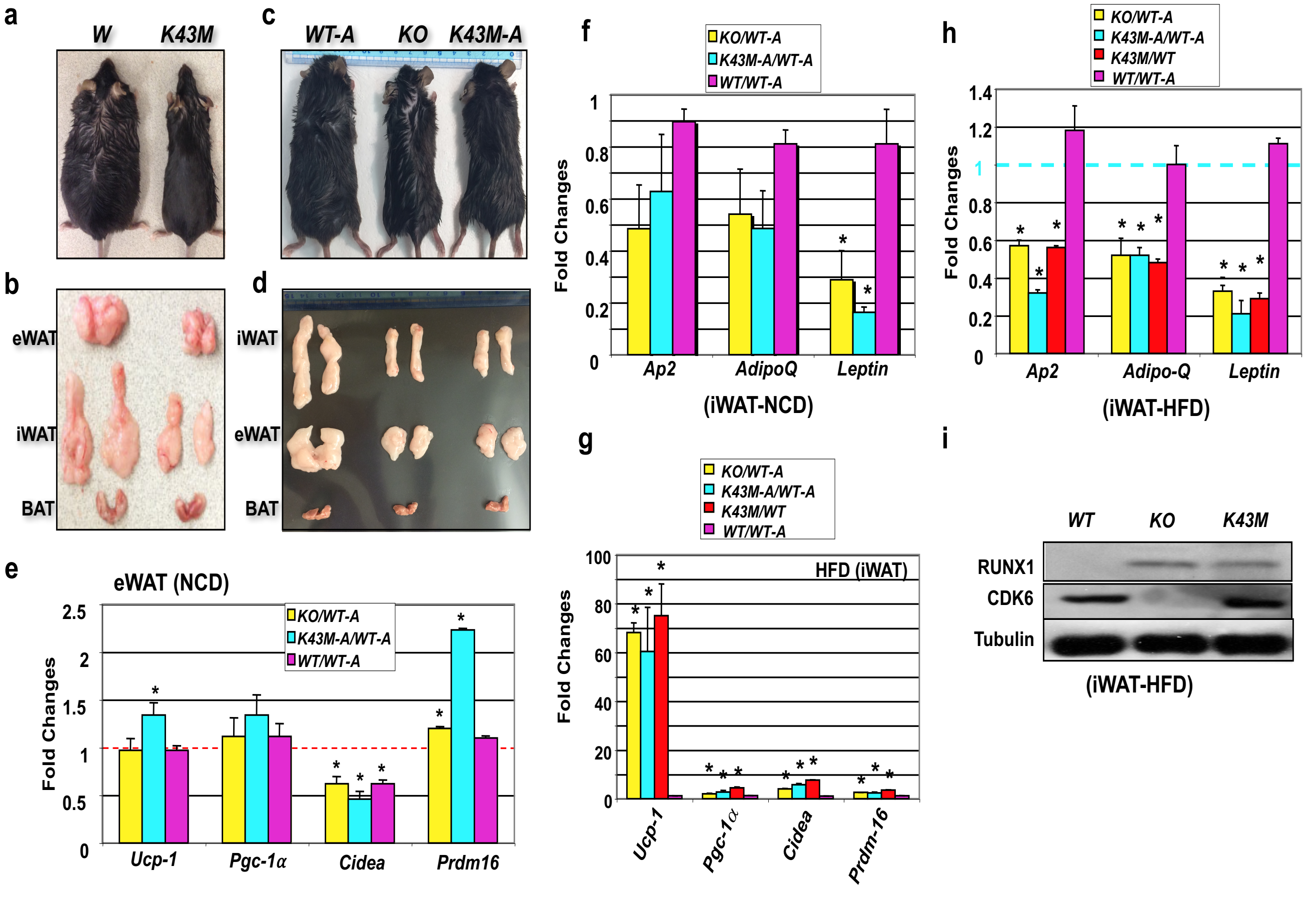

Supplementary Figure 3. *Loss of CDK6 protein or kinase activity in germline or adipocytes in mice* ameliorates diet-induced obesity (DIO), whereas re-repression of CDK6 in *adipocytes in mice resumed*

the susceptibility to DIO. (**a,c**) Representative photographs of *WT*, *K43M*, *WT-A*, *KO*, and *K43M-A* mice after 14 weeks on HFD. (**b,d**) Appearance of a close view of the iWAT, eWAT, and BAT from the mice indicated after 14 weeks on HFD. (**e,g**) Relative mRNA expression levels of BAT-specific markers (*Ucp-1*, *Pgc-1α, Cidea, and Prdm16) in* eWAT of NCD (e) or HFD-fed (g) mice. (**f,h***)* WAT-specific markers (*Ap2*, *Leptin, and AdipoQ)* of iWAT of NCD-fed (**f**) or HFD-fed (**h**) mice indicated. For e-h, data shown are fold changes of mRNA normalized to the control *WT or WT-A,* which is arbitrarily set to 1 unit. $^*p < 0.05$, (n=6), *vs WT*, or *vs WT-A,* t-test. (**i**) Immunoblots showing the expression levels of RUNX1 and CDK6 protein of iWAT under HFD.

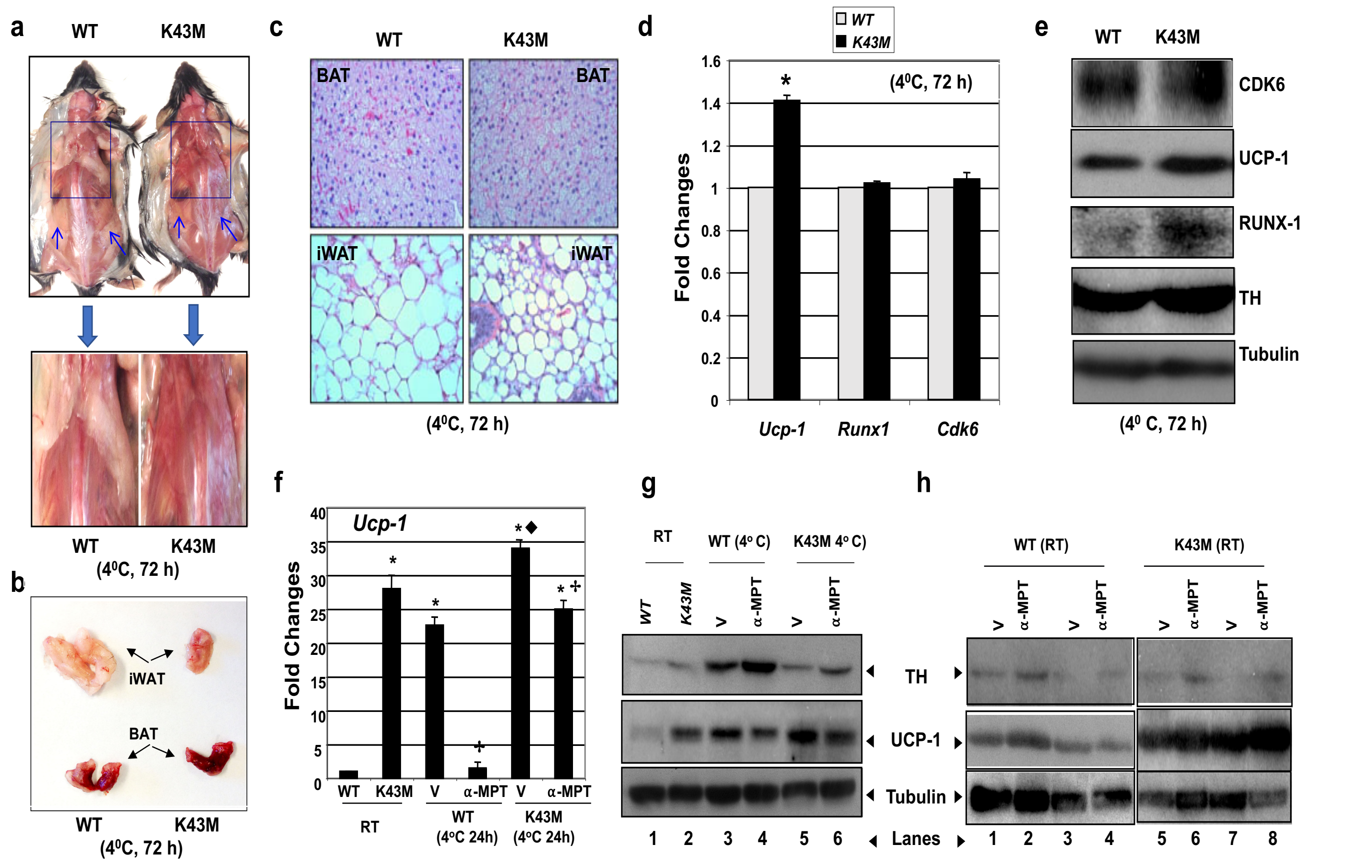

Supplementary Figure 4. Increased adrenergic stimulation is not the major determinant of white fat browning in *K43M* mice. (**a**) Representative dorsal view of chow fed *WT* and *K43M* adult mice exposed to 4°C for 72h. The bottom panel is enlarged image of indicated square areas. (**b**) Representative photograph of iWAT and BAT from chow fed WT and *K43M* adult mice exposed to 4°C for 72h. (**c**) H&E staining of BAT and iWAT for representative WT and *K43M* mice after 72h cold exposure. (**d**) Expression level of *Ucp-1, Runx1, and Cdk6* mRNAs in iWAT from *WT* or *K43M* mice following 72-hour cold

exposure. Data shown are fold changes of mRNA from each condition normalized to the control mice (*WT*), which is arbitrarily set to 1 unit. *$p < 0.05$ (n=6), *vs WT* (n=6), t-test. (**e**) Immunoblots showing the expression levels of indicated proteins of iWAT under cold exposure for 72hr. (**f**) Expression level of *Ucp-1* in the presence of absence of α–MPT (150 mg/kg of BW) under cold exposure for 24hr. Data shown are fold changes of mRNA from each condition normalized to the control mice (*WT*) under RT, which is arbitrarily set to 1 unit. *$p < 0.05$, *vs WT* (n=6), t-test. ◆$p < 0.05$ (n=6), t-test, *K43M* at 4^0C *vs K43M at* RT. ^+p <0.05 *vs Vehicle (V: H2O),* n=6, t-test. (**g,h**) Immunoblots showing the expression levels of indicated proteins of iWAT under cold exposure or RT for 24hr.

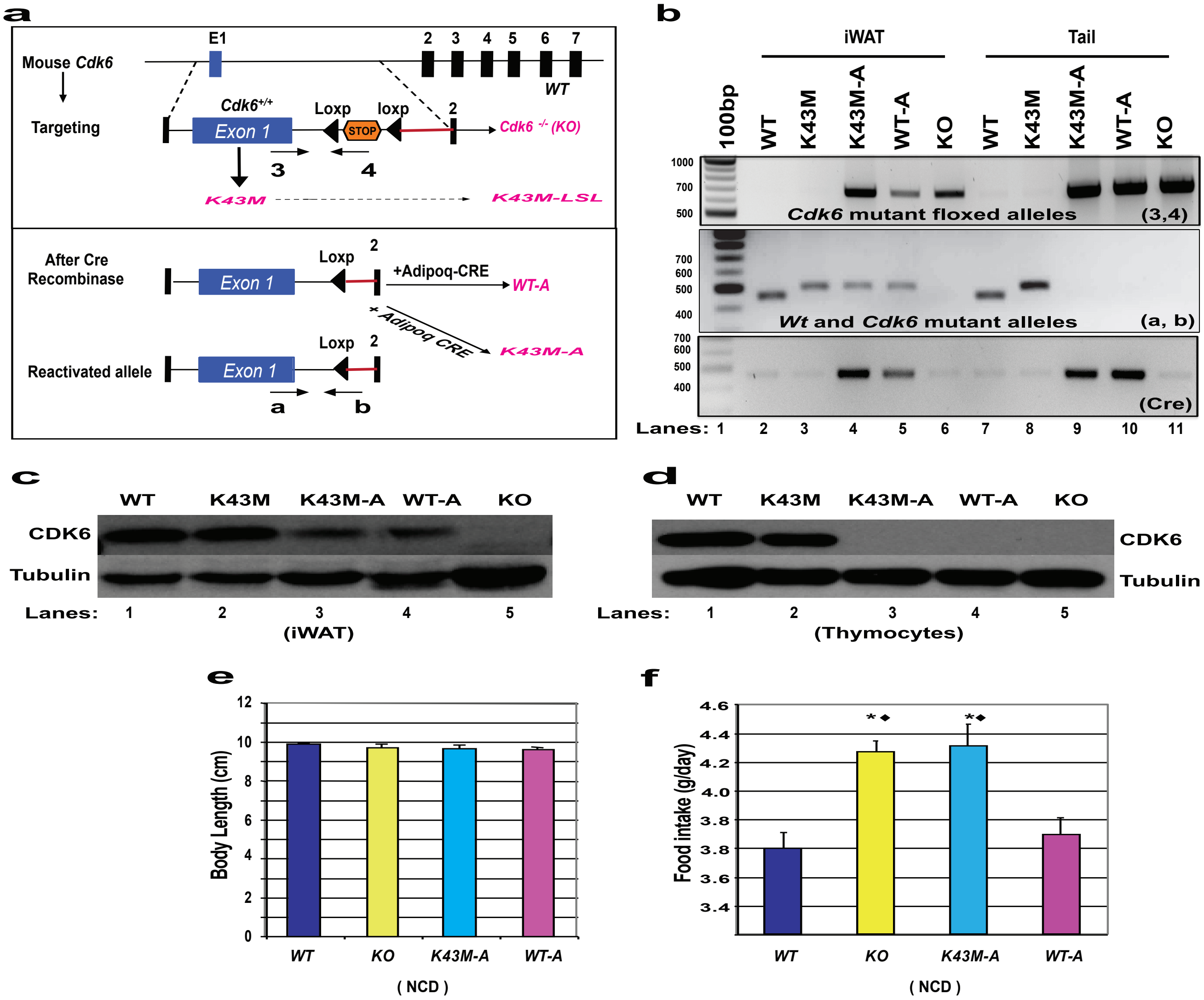

Supplementary Figure 5. Characterization of resultant mice with adipocytes-specific expression of WT/K43M. (**a**) The strategy used for making *Cdk6* mutant mice with adipocytes-specific expression of

WT/K43M. *Cdk6*$^{-/-}$ mice were made as described in Figure S2 (**a**, top panel). The schematic diagram underneath "Targeting allele" showed that Primers 3 (on exon 1) and 4 (on Stop cassette), which flanks the LSL cassette to detect the presence of LSL. Upon excision of the cassette by Adipoq-CRE, which is expressed in mature adipocytes, the CRE-reactivated *WT/K43M* alleles will express CDK6/K43M protein in mature adipocytes (*WT-A* or *K43M-A mice*) (**a**, bottom panel). The schematic diagram underneath "Reactivated allele" showed that Primers a (on exon 1) and b (on intron 1), which flanks one Lox-P site after recombination by CRE. (**b**) PCR-based genotyping confirmed DNA recombination in the adipose tissues (e.g. iWAT, lanes 4 and 5) of *WT-A and K43M-A* mice but not in the tail DNA (lanes 9 and 10). DNA isolated from iWAT or tails of *WT* (lanes 2 and 7), *K43M* (lanes 3 and 8), *K43M-A (*lanes 4 and 9), *WT-A* (lanes 5 and 10), *KO* (lanes 6 and 11) mice using specific primers as indicated to detect the presence of LSL cassette (~700bp, p:3,4) or deletion of LSL (with one 34 bp LoxP site in the intron 1, ~500 bp, p:a,b), and CRE gene (bottom panel, p: CRE-R,F). We can detect ~700bp (LSL) fragment in *WT-A* and *K43M-A* mice, which may come from pre-adipocytes and other non-adipocyte cells in the iWAT since Adipoq-CRE is only expressed in mature adipocytes. It is also possible that the presence of LSL is due to the partial penetrating recombination. (**c** and **d**) CDK6 protein levels of iWAT (C) and thymocytes (d) under NCD. α-Tubulin was used as an internal control. (**e**) Body length of different mice indicated (n=10 for each strain). (f) Food intake of different mice indicated. *$p < 0.05$, (n=6), *vs WT*, t-test. ◆$p < 0.05$, t-test, *vs WT-A.*

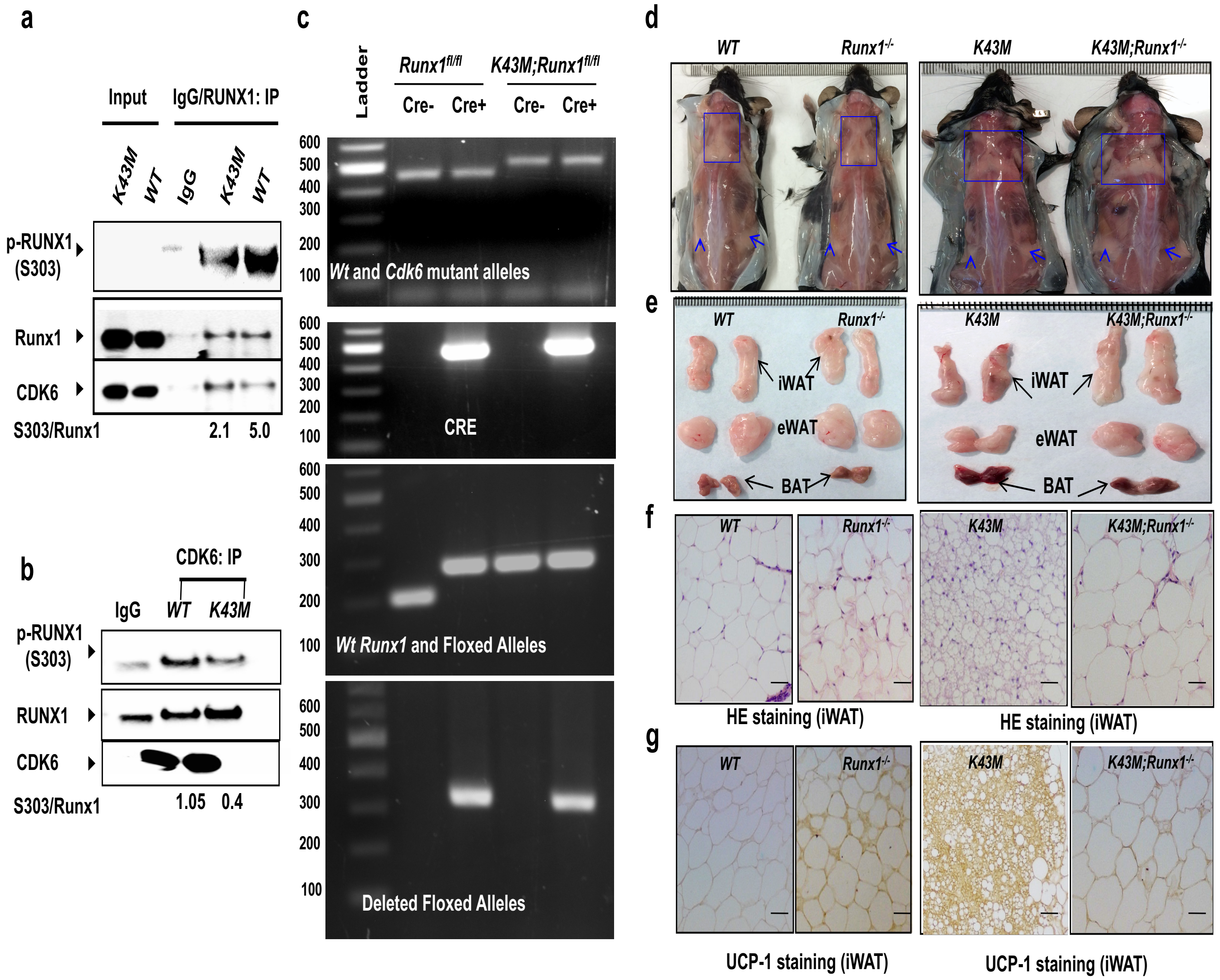

Supplementary Figure 6. Ablation of RUNX1 on K43M background restores the ability of K43M mice to gain weight and reverses white fat browning, improved GTT observed in K43M mice under NCD. (**a**) Co-Immunoprecipitation showing that CDK6 interact with RUNX1. RUNX1 was immuno-precipitated with antibody from extracts of iWAT from *WT* and *K43M* mice, and the blots were probed with the indicated antibodies against p-RUNX1(S303), total RUNX1, and CDK6. As a negative control, same amount of *WT* cell lysates was immuno-precipitated with normal mouse IgG. “Input” represents the amount of extract (50 μg) subjected to immunoblotting. The ratios of S303/RUNX1 was indicated below the panels. (**b**) Co-Immunoprecipitation showing that CDK6 interact with RUNX1. CDK6 was immuno-precipitated with antibody from extracts of iWAT from *WT* and *K43M* mice, and the blots were probed with the indicated antibodies against p-RUNX1(S303), total RUNX1, and CDK6. As a negative control, same amount of *WT*

cell lysates was immuno-precipitated with normal mouse IgG. The ratios of S303/RUNX1 was indicated below the panels. (**c**) PCR-based genotyping confirmed DNA recombination in the adipose tissues. Four panels showed (1) WT alleles (~466bp) and the deletion of LSL by using primers a and b as indicated in Supplementary Fig. 4b (with one 34 bp loxp site in the intron 1, ~500 bp, p:a,b); (2) CRE expression; (3) *WT* and Floxed *Runx1* alleles[31]; (4) Deleted Floxed Runx1 alleles[31]. (**d**) Appearance of male posterior-subcutaneous WAT (sWAT), emphasized with blue squares and arrows. (**e**) Appearance of a close view of the iWAT, eWAT, and BAT from the mice indicated. (**f**) Representative light microscopic images of H&E-stained sections of iWAT (n=6) from male mice indicated (scale bars: 100 μm). (**g**) Representative images of UCP-1 staining (n=6) of iWAT from mice indicated at 12 weeks of age (scale bars: 100 μm).

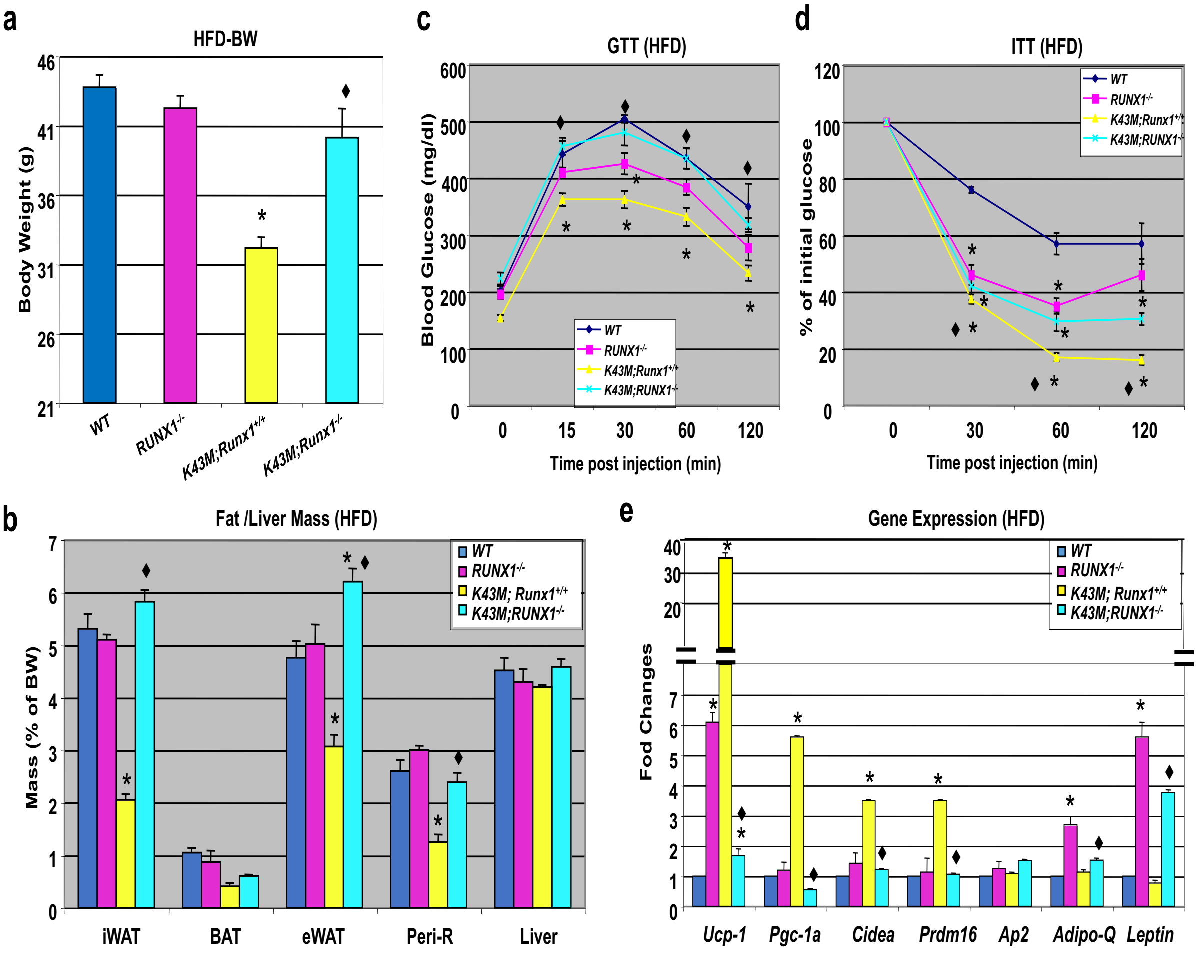

Supplementary Figure 7. Ablation of RUNX1 in K43M mice restores weight gain, while reversing white fat browning, improved GTT and ITT under HFD. (**a**) Body weight of age-matched male mice (18-week

old) on HFD. *$p < 0.05$, (n=6), *vs WT*, t-test. ◆$p < 0.05$, t-test, *K43M;Runx1$^{-/-}$ vs K43M.* (**b**) Mass of various fat pads was normalized to body weight of male mice on HFD at age of 18 weeks. No significant changes were observed in the masses of BAT and livers among mice indicated under HFD. Data shown are mean ± S.E. (n=10 for each group), *$p < 0.05$, t-test, *vs WT*. ◆$p < 0.05$, t-test, *K43M;Runx1$^{-/-}$ vs K43M*. (**c**) GTT after 14 weeks on HFD. (**d**) ITT after 14 weeks on HFD. For **c** and **d**, n=10 for each group, *$p < 0.05$, t-test, *vs WT*, ◆$p < 0.05$, t-test, *K43M;Runx1$^{-/-}$ vs K43M.* (**e**) Relative mRNA expression levels of BAT-specific markers and WAT-specific markers of iWAT tissues from mice indicated. Data shown are fold changes of mRNA normalized to the control *WT,* which is arbitrarily set to 1 unit. *$p <$ 0.05, (n=6), *vs WT* control, t-test. ◆$p < 0.05$, t-test, *K43M;Runx1$^{-/-}$ vs K43M.*

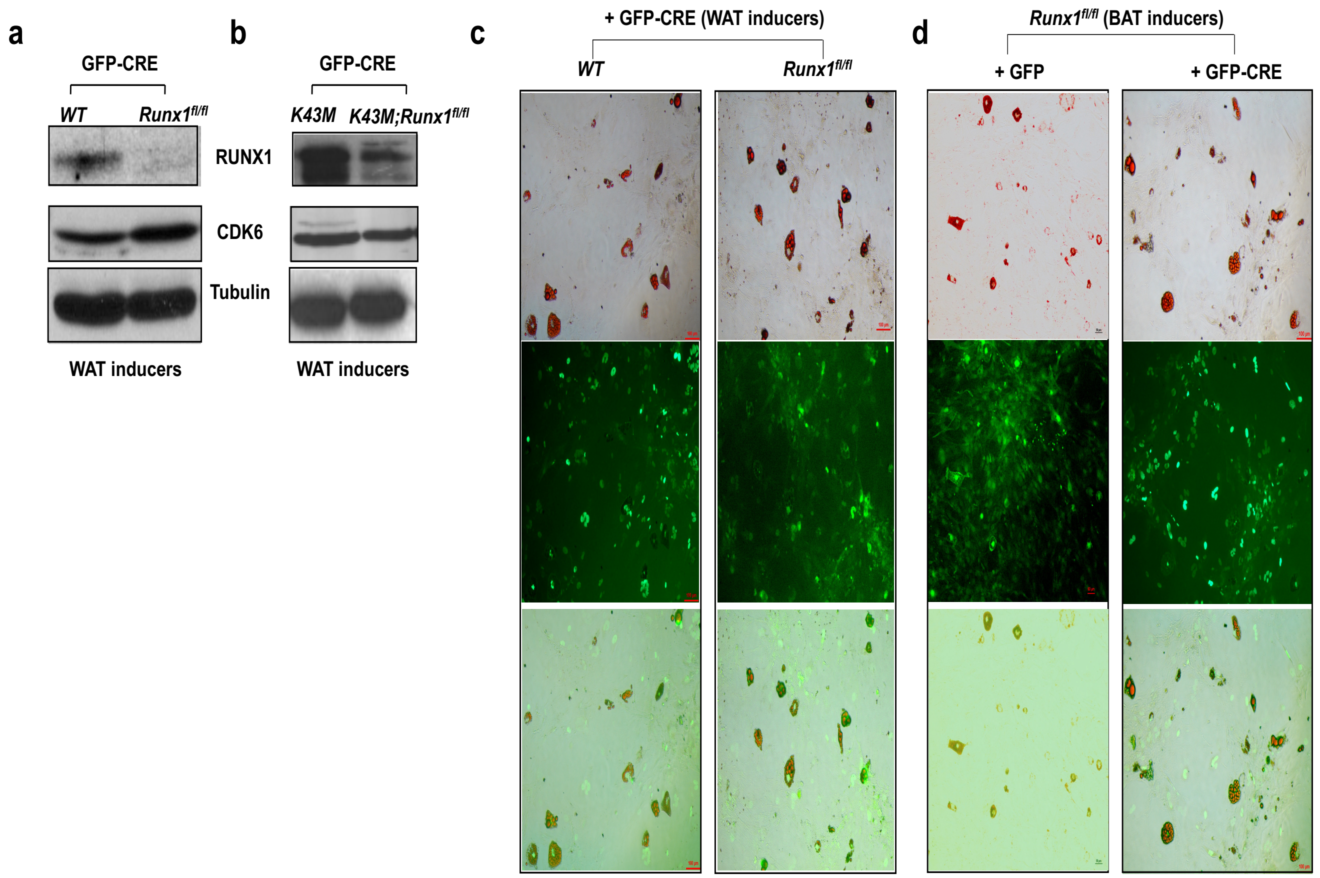

Supplementary Figure 8. Ablation of RUNX1 in *WT* precursors does not alter the capacity of differentiation. **(a,b)** Immunoblots of the indicated protein levels in the differentiated cells from 50 μg of

cell lysates as indicated after 7 days in the presence of WAT inducers. α-tubulin is used as internal loading control. (**c,d**) Fluorescent photomicrographs of differentiated cells from *WT*/*Runx1$^{fl/fl}$* + GFP-Cre (**c**, WAT inducers) or *Runx1$^{fl/fl}$* + GFP/GFP-Cre (**d**) in the presence of BAT inducers. Red fluorescence indicates the Oil Red O staining. Green fluorescence indicates the expression of GFP/GFP-Cre. The bottom panels of images (**c,d**) are merged images. Scale bar: 100 μm.

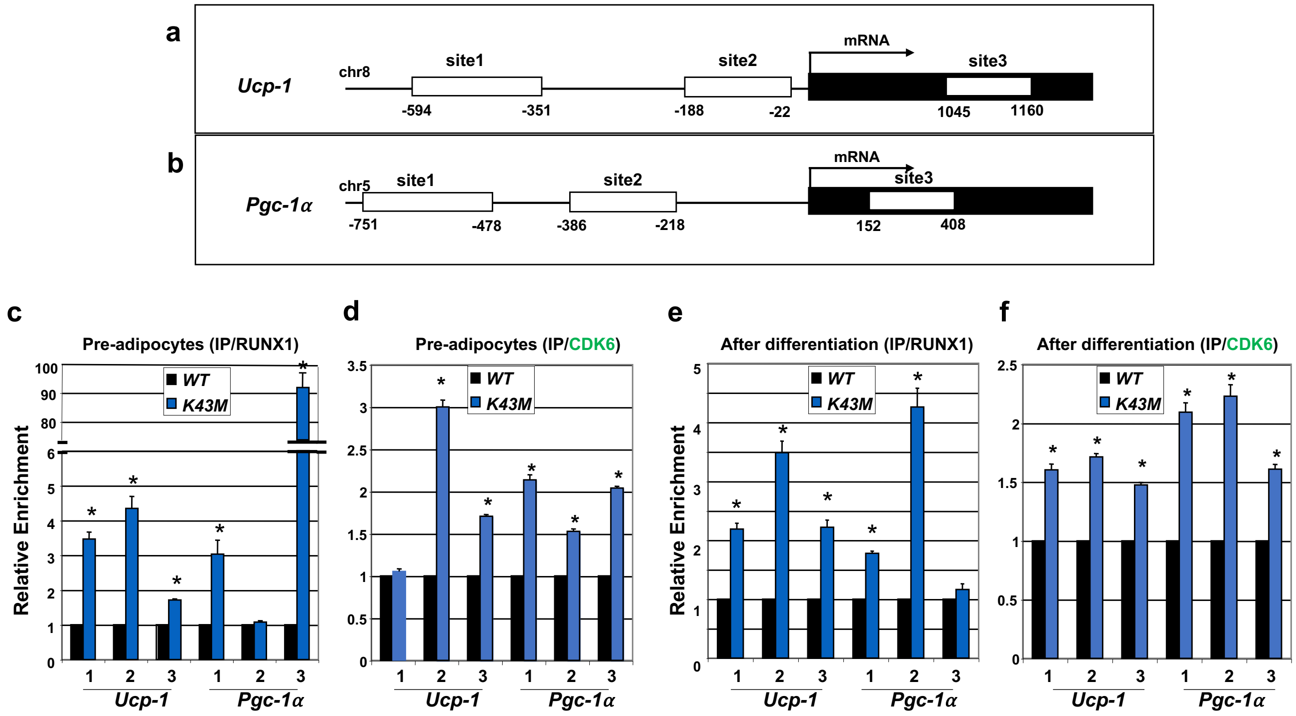

Supplementary Figure 9. RUNX1 binds to RUNX1-consensus-binding sites in the mouse *Ucp-1* and *Pgc-1α* genes. (**a,b**) Schematic diagram of location of three candidate RUNX1 binding sites. Site 1 and 2 are in the proximal promoter regions of *Ucp-1/PGC-1α gene.* Site 3 is on the exon 1 of *Ucp-1/Pgc-1α* gene*.* The numbers below show the positions of primers pair used in RUNX1 ChIP assay*.* **(c-f)** ChIP-qPCR analysis of the binding of RUNX1 to the indicated regulatory regions of *Ucp1* and *Pcg1α* gene in the preadipocytes (**c,d**) or differentiated cells (**e,f**) by using either RUNX1 or CDK6 antibodies. Data shown are mean ± S.E. (n=3 for each group), *$p < 0.05$, t-test, *vs WT*.

(B) SUPPLEMENTARY TABLE1. Mouse qRT-PCR Primer Sequences

Gene	Forward Primer	Reverse Primer
Runx1	TTT CGC AGA GCG GTG AAA GA	GCA CTG TGG ATA TGA AGG AA
Ap2	ACA CCG AGA TTT CCT TCA AAC TG	CCA TCT AGG GTT ATG ATG CTC TTC A
Adiponectin	GCA CTG GCA AGT TCT ACT GCA A	GTA GGT GAA GAG AAC GGC CTT GT
Leptin	CTG CCC CCC AGT TTG ATG	GCC AGG CTG CCA GAA TTG
36B4	AGA TGC AGC AGA TCC GCA T	GTT CTT GCC CAT CAG CAC C
Ucp-1 site1	GAC CAC ACG ATG CAC TCA CT	GAT GAA CGT CCC AGT CCC G
Ucp-1 site2	CTGGTGCCAAATCAGAGGTG	CTA TAT AGC CCC TTG CCG GAG
Ucp-1 site3	CGC CTT CAG ATC CAA GGT GA	AGA CCG CTG TAC AGT TTC GG
Pgc-1α site 1	GCA AGG CAA ACT GCA GTA ACA	GAG TGT TTG AAG GCA ACA CCCC
Pgc-1α site 2	GCT GAG TCT GGG GCT ACT TG	GCC ACC AAC TCT AAA CCG GA
Pgc-1α site 3	TGT GCA GCC AAG ACT CTG TA	GTG TCT CTG TGA GAA CCG CT

(C) SUPPLEMENTARY NOTES (uncropped scans of the most important western blots)

Figure 2I (iWAT)

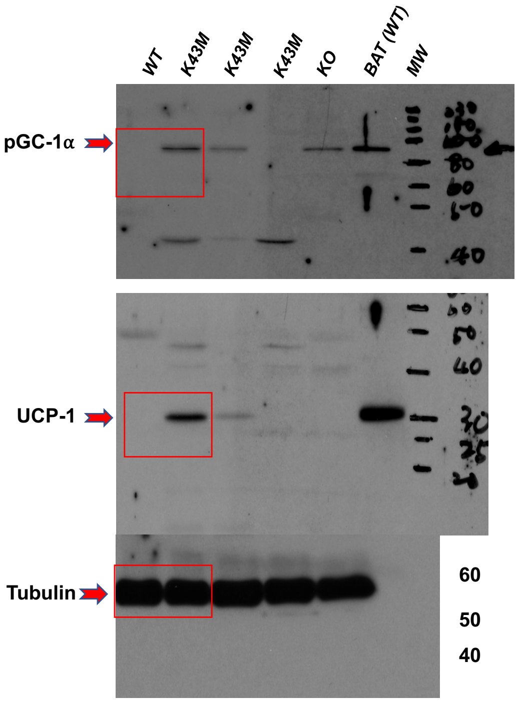

Figure 2I (eWAT)

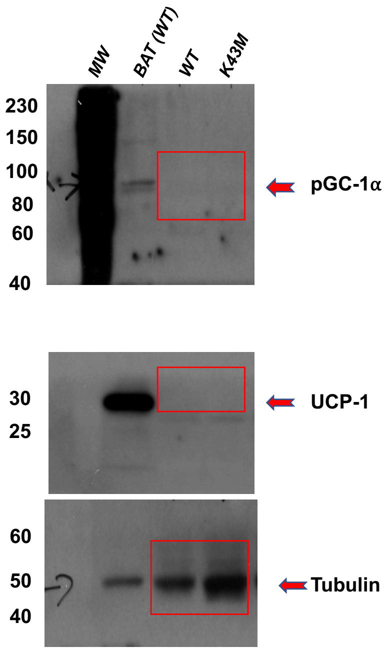

Figure 4f (iWAT)

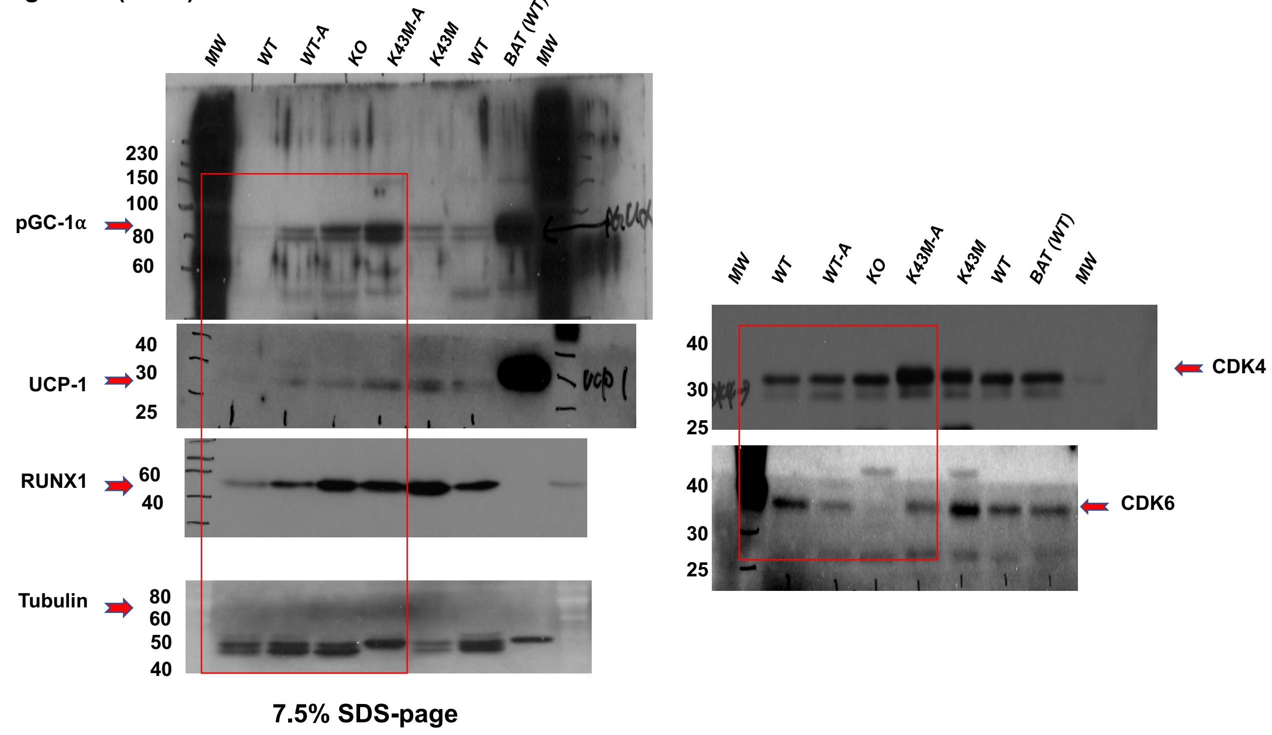

Figure 4f (eWAT)

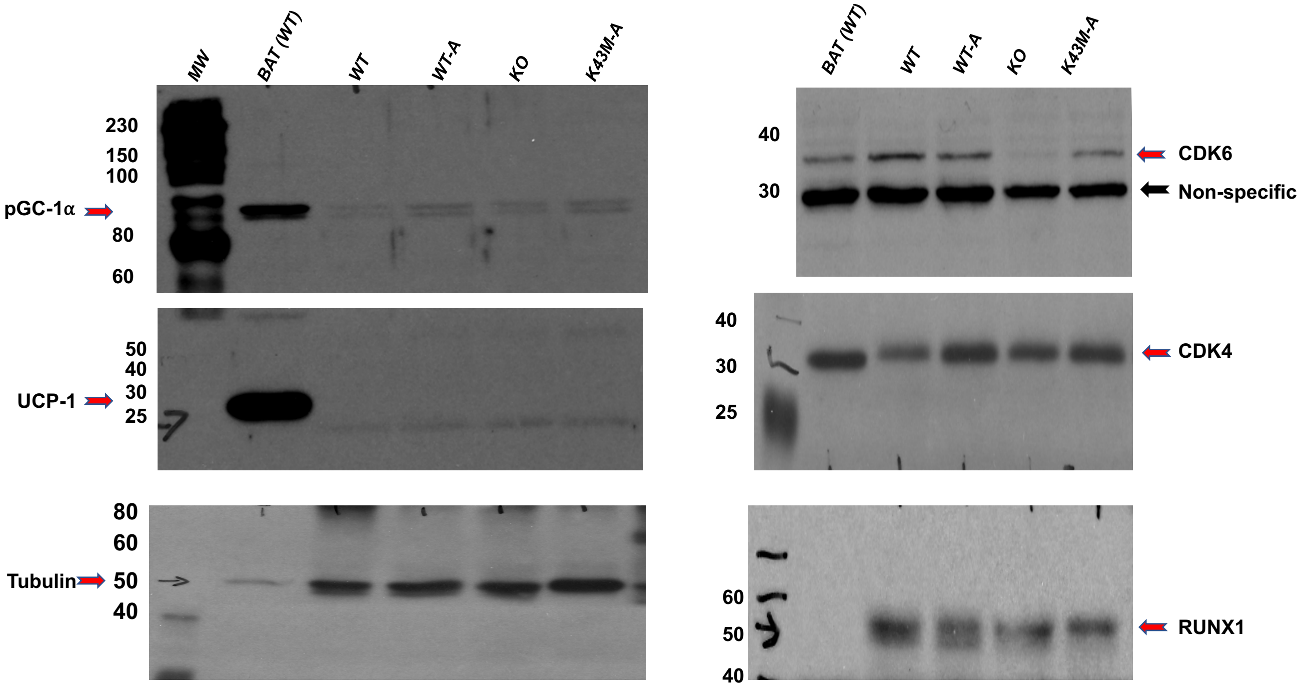

Figure 7f

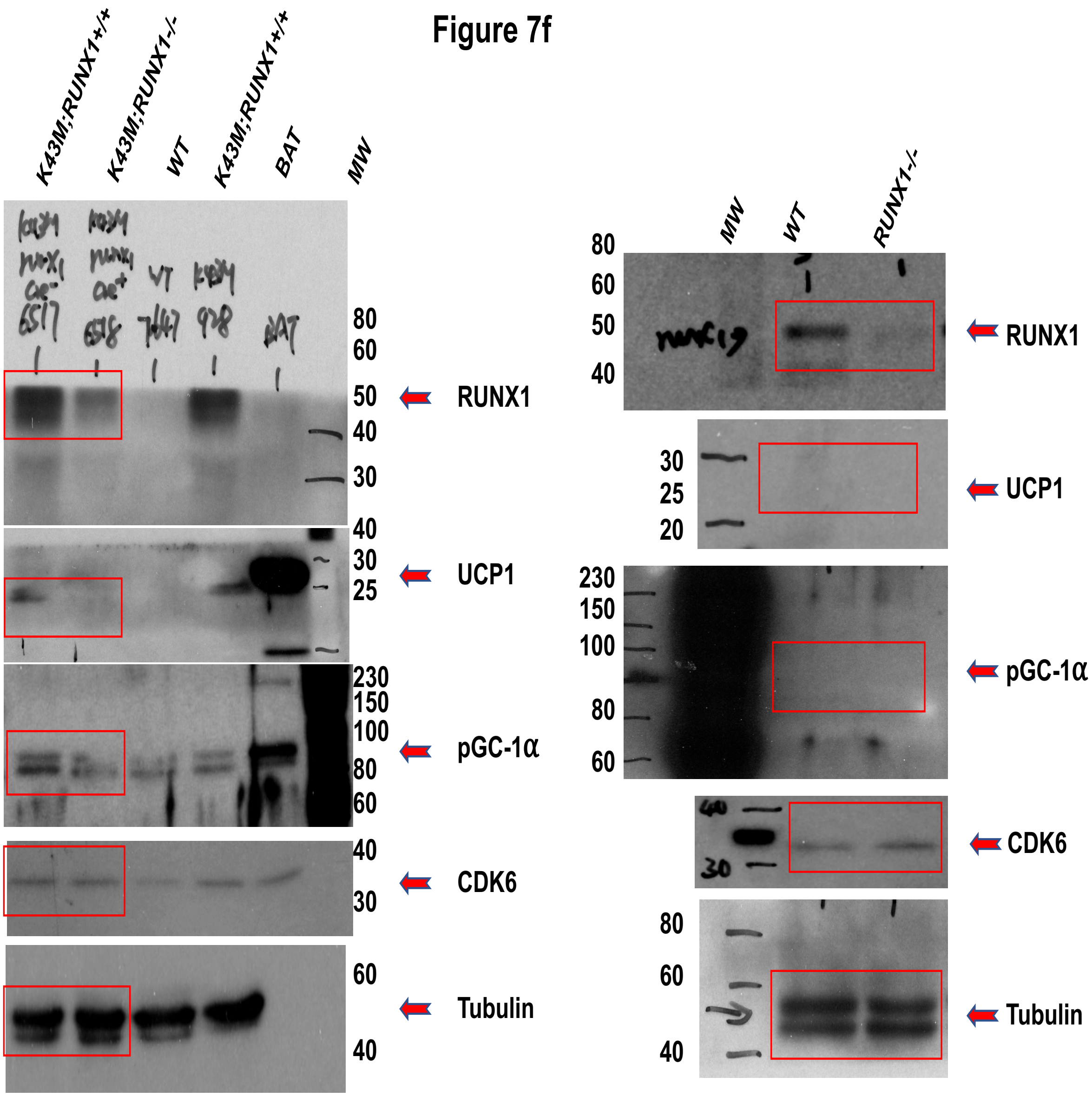
K43M;RUNX1+/+
K43M;RUNX1-/-
WT
K43M;RUNX1+/+
BAT
MW
RUNX1
UCP1
pGC-1α
CDK6
Tubulin
80
60
50
40
30
25
230
150
100
MW
WT
RUNX1-/-
20
